# Supplementary material for: Highly robust supramolecular polymer networks crosslinked by a tiny amount of metallacycles
Source: Nat Commun. 2024 Apr 9;15:3050. doi: 10.1038/s41467-024-47333-1 (PMC11004166; doi:10.1038/s41467-024-47333-1)
Supplement: Supplementary file 1 — Supplementary Information [file 41467_2024_47333_MOESM1_ESM.pdf]

Supplementary Information for

Highly robust supramolecular polymer networks crosslinked by a tiny amount of metallacycles

Lang He,[1] Yu Jiang,[2] Jialin Wei,[1] Zibin Zhang,[1] Tao Hong,[2] Zhiqiang Ren,[3] Jianying Huang,*[,2] Feihe Huang,*[4,5] Peter J. Stang,*[,6] and Shijun Li*[,1]

[1]College of Material, Chemistry and Chemical Engineering, Key Laboratory of Organosilicon Chemistry and Material Technology of Ministry of Education, Hangzhou Normal University, Hangzhou 311121, P. R. China.

[2]College of Food Science and Biotechnology, Zhejiang Gongshang University, Hangzhou 310018, P. R. China.

[3]School of Materials Science and Engineering, Peking University, Beijing 100871, P. R. China.

[4]Stoddart Institute of Molecular Science, Department of Chemistry, Zhejiang University, Hangzhou 310058, P. R. China.

[5]Zhejiang-Israel Joint Laboratory of Self-Assembling Functional Materials, ZJU-Hangzhou Global Scientific and Technological Innovation Center, Zhejiang University, Hangzhou 311215, P. R. China.

[6]Department of Chemistry, University of Utah, 315 South 1400 East, Room 2020, Salt Lake City, Utah 84112, United States.

*Corresponding authors. E-mails: huangjy@zjgsu.edu.cn; fhuang@zju.edu.cn; stang@chem.utah.edu; l_shijun@hznu.edu.cn

Table of Contents

1.	**Supplementary Materials and methods**	**S2**
2.	**Synthesis of monomer M-1**	**S4**
3.	**Synthesis of 60° acceptor 7 and 120° acceptor 8**	**S20**
4.	**Synthesis of norbornene-functionalized rhomboid 9**	**S23**
5.	**Synthesis of norbornene-functionalized hexagon 10**	**S27**
6.	**Preparation of covalent polymers CPs**	**S30**
7.	**Preparation of metallacycle-crosslinked polymer networks CP-*n*Rs**	**S37**
8.	**Preparation of metallacycle-crosslinked polymer networks CP-*n*Hs**	**S38**
9.	**TGA of metallacycle-crosslinked polymer networks**	**S39**
10.	**Possible incomplete assemblies during the self-assembly of dipyridine units in polymers**	**S40**
11.	**Preparation of control ^{con}CP-3R and ^{con}CP-3H**	**S41**
12.	**Mechanical properties of CP-3, CP-3R, CP-4R, CP-3H, CP-4H and the controls**	**S42**
13	**The storage modulus of CP-3, CP-3R and CP-3H from DMA**	**S43**
14.	**The swelling of CP-3R and CP-3H, and the dissolution of CP-3**	**S44**
15.	**Morphologies of the xerogels CP-3R and CP-3H**	**S45**
16.	**Stimuli-responsive properties of metallacycle-crosslinked polymer networks**	**S46**
17.	**Mechanical properties of CP-3R + Br^-, CP-3R + Br^- + Ag^+, CP-3H + Br^- and CP-3H + Br^-+ Ag^+**	**S47**
18.	**Single-crystal X-ray structure of 9**	**S48**
19.	**Supplementary References**	**S50**

1. Supplementary Materials and methods

All reagents and solvents were commercially available and used as supplied unless otherwise stated. Compounds **1**[1], **7**[2], **8**[3], **M-2**[4], **11**[2], and **12**[3] were prepared according to the established methods, the detailed synthetic procedures were also provided in the following text. For these reported compounds, ^{1}H NMR spectra were measured and compared with those in the literatures to prove their structures. Deuterated solvents were purchased from Cambridge Isotope Laboratory. All reactions were performed under ambient laboratory conditions, and no precautions were taken to exclude atmospheric moisture unless otherwise specified. The NMR spectra were collected on a Bruker AVANCE DMX-500 spectrometer and chemical shifts were reported relative to internal standard tetramethylsilane (TMS) at 0.00 ppm or the residual solvent signals. Electrospray ionization mass spectra (ESIMS) were obtained on an Agilent 1290-6530 UPLC-Q-TOF spectrometer using electrospray ionization. Suitable crystals were selected and measured on a Bruker APEX-II CCD diffractometer. The crystal was kept at 170.0 K during data collection. The structure was solved with the ShelXT structure solution program using Intrinsic Phasing and refined with the ShelXL refinement package using Least Squares minimization. Single crystal X-ray data of the rhomboid **9** have been deposited in Cambridge Crystallographic Data Centre (CCDC). Its deposition number is 2297067.

Gel permeation chromatograph (GPC) was obtained on an HLC8320 GPC (TOSOH, Japan) instrument using tetrahydrofuran (THF) as eluent with polystyrene standards. The thermal stability analysis was conducted using a TA Instruments Q500 thermogravimetric analyzer (TGA) under nitrogen atmosphere. Each sample (~5 mg) was heated from 50 to 650 °C with a rate of 20 °C/min. Transition temperatures of materials were determined on a TA Instruments Q2000 differential scanning calorimetry (DSC) under nitrogen atmosphere. Dynamic thermomechanical analysis (DMA) was carried out with a dynamic mechanical thermal analyzer (DMA-TAQ800) in the tensile mode at a frequency of 1.0 Hz. Cryofractured surfaces of polymers were sputter-coated with a thin layer of gold and then examined using a scanning electron microscope (FE-SEM, Hitachi S-4800), operating at an accelerating voltage of 3.0 kV.

Mechanical tests: The mechanical properties of the polymers were measured using an Instron 5966 machine in air at room temperature. The tensile curves were measured at a constant speed of 100 mm/min. Young’s modulus was determined from the initial slope of the stress-strain curve. Toughness was obtained by integrating the area under stress-strain curve. Energy dissipation was calculated by integrating the area encompassed by the cyclic tensile curve. Damping capacity was defined as the ratio of the dissipated energy (the area encompassed by the loading and unloading curves) to the loading energy (the area encompassed by the loading curve).

2. Synthesis of monomer M-1

Synthesis of compound 1

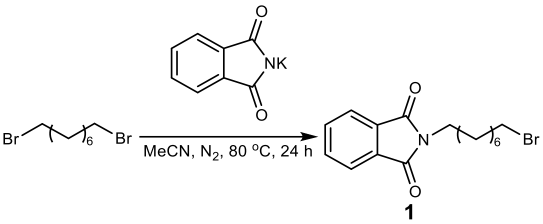

Potassium phthalimide (3.00 g, 16.20 mmol), 1,8-dibromooctane (8.81 g, 32.40 mmol) and 100 mL of MeCN were added to a 250 mL round-bottomed flask. After heating at 80 °C under N_2 atmosphere for 24 h, the solution was concentrated and the resulting residue was purified by flash column chromatography (dichloromethane/petroleum ether, 1:1 *v*/*v*) to afford compound **1** as a white solid (4.08 g, 74%). ^1H NMR (500 MHz, $CDCl_3$, 298 K) δ 7.88–7.81 (m, 2H), 7.75–7.68 (m, 2H), 3.72–3.65 (m, 2H), 3.39 (t, J = 7 Hz, 2H), 1.87–1.80 (m, 2H), 1.70–1.64 (m, 2H), 1.42 (dt, J = 15, 7 Hz, 2H), 1.37–1.29 (m, 6H).

Synthesis of compound 2

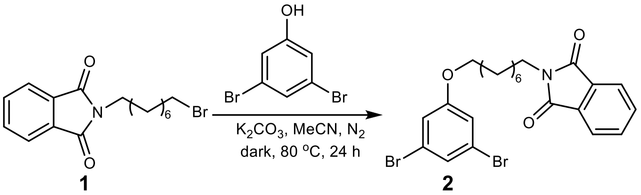

Compound **1** (2.00 g, 5.91 mmol), 3,5-dibromophenol (2.23 g, 8.87 mmol), potassium carbonate (2.45 g, 17.74 mmol), and 100 mL of MeCN were added to a 250 mL round-bottomed flask. After heating at reflux under N_2 for 24 h, the solvent was removed and CH_2Cl_2 was added. The mixture was washed with water and brine, and then dried over anhydrous Na_2SO_4. The solution was concentrated and the resulting residue was purified by flash column chromatography (dichloromethane/petroleum ether, 1:3 *v*/*v*) to afford compound **2** as a white solid (2.98 g, 99%). Mp 98.7–99.6 °C. The ^1H NMR spectrum of compound **2** is shown in Supplementary Fig. 1. ^1H NMR (500 MHz, $CDCl_3$, 298 K) δ 7.86–7.81 (m, 2H), 7.73–7.67 (m, 2H), 7.21 (t, J = 2 Hz, 1H), 6.96 (d, J = 2 Hz, 2H), 3.89 (t, J = 7 Hz, 2H), 3.68 (t, J=7 Hz,

2H), 1.77 – 1.70 (m, 2H), 1.70 – 1.65 (m, 2H), 1.45–1.38 (m, 2H), 1.37–1.31 (m, 6H). The ^{13}C NMR spectrum of compound **2** is shown in Supplementary Fig. 2. ^{13}C NMR (126 MHz, $CDCl_3$, 298 K) δ 168.6, 160.5, 134.0, 132.3, 126.3, 123.2, 117.1, 68.7, 38.1, 29.2, 29.1, 29.0, 28.7, 26.8, 25.9. HRMS (ESI/Q-TOF) of compound **2** is shown in Supplementary Fig. 3. *m*/*z*: $[M + Na]^+$ calcd for $C_{22}H_{23}Br_2NNaO_3$, 529.9937; found, 529.9951.

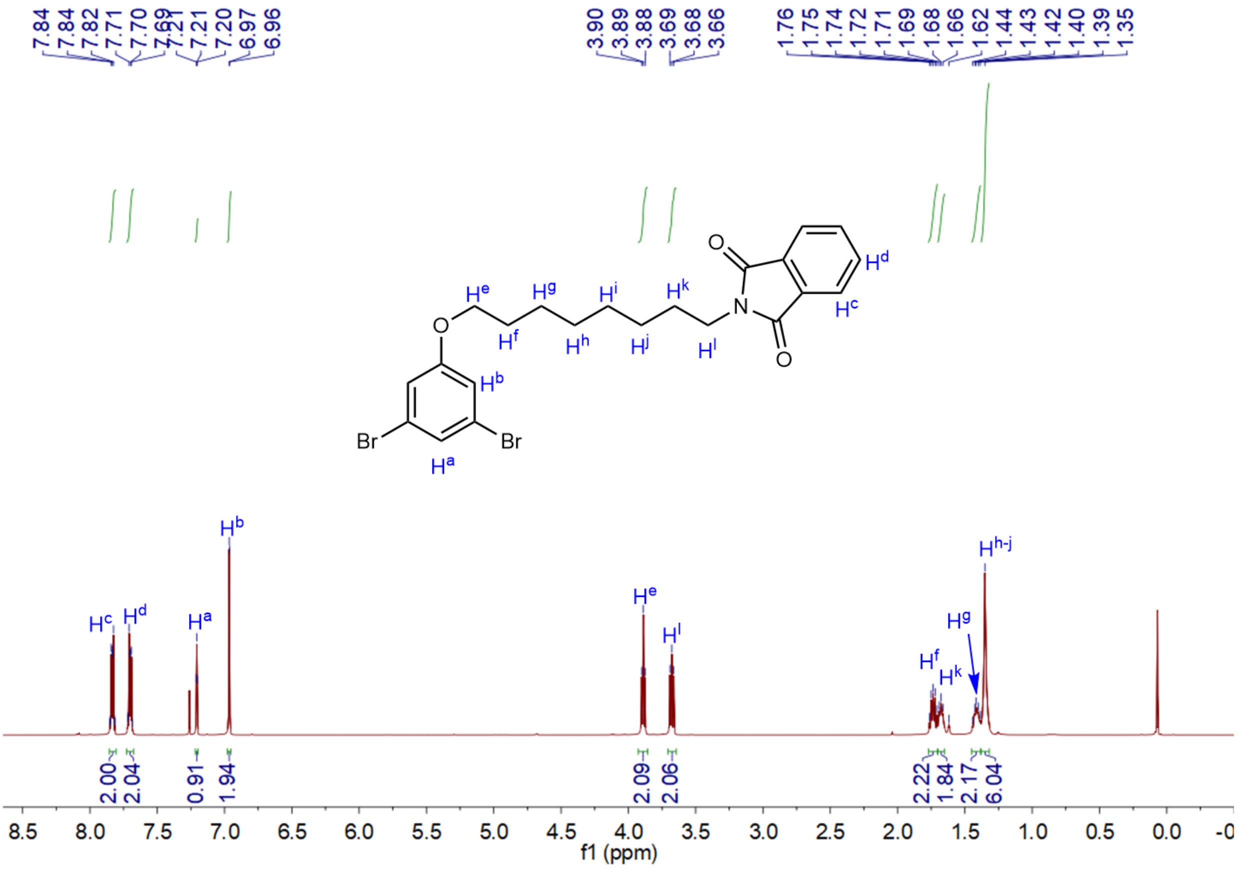

Supplementary Fig. 1 ^{1}H NMR spectrum (500 MHz, $CDCl_3$, 298 k) of compound **2**.

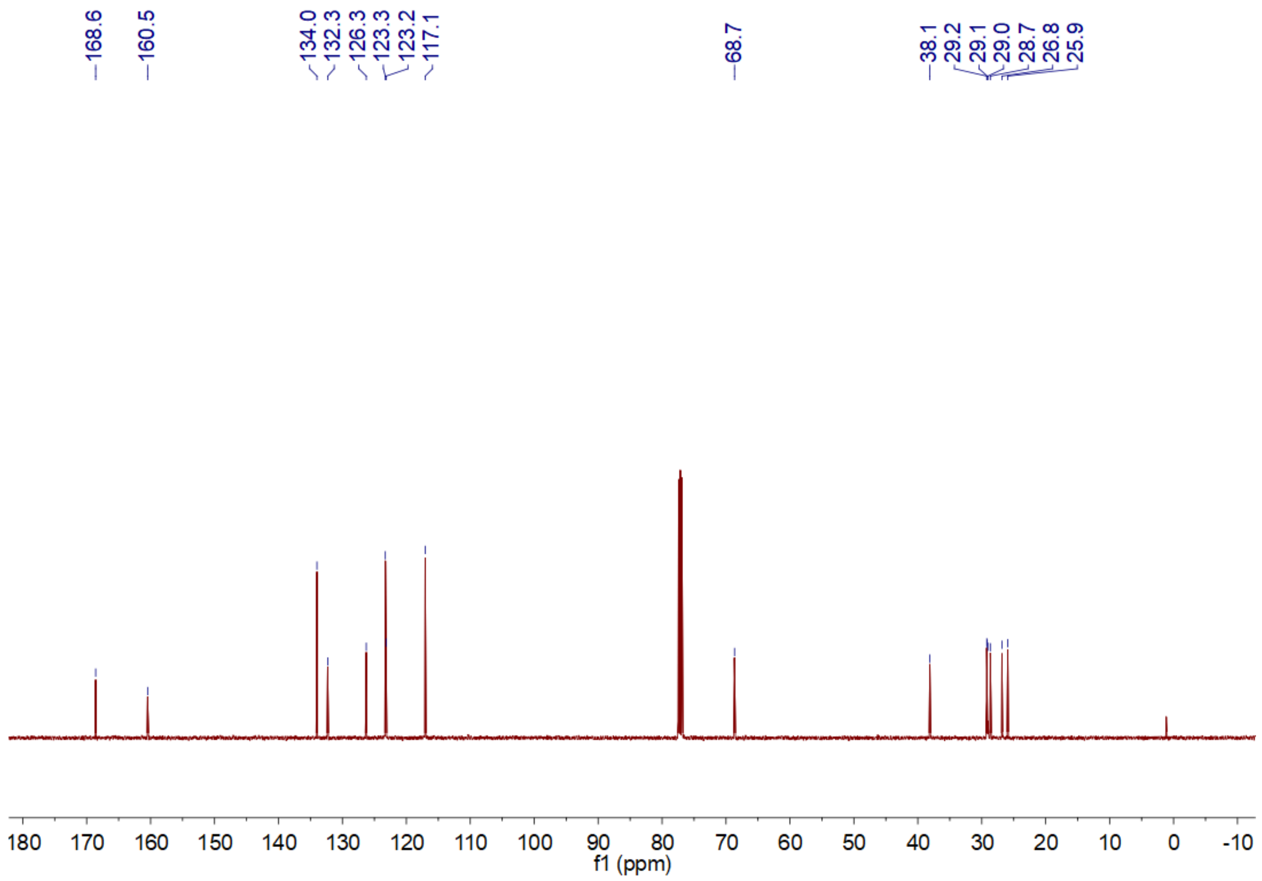

Supplementary Fig. 2 ^{13}C NMR spectrum (500 MHz, $CDCl_3$, 298 K) of compound **2**.

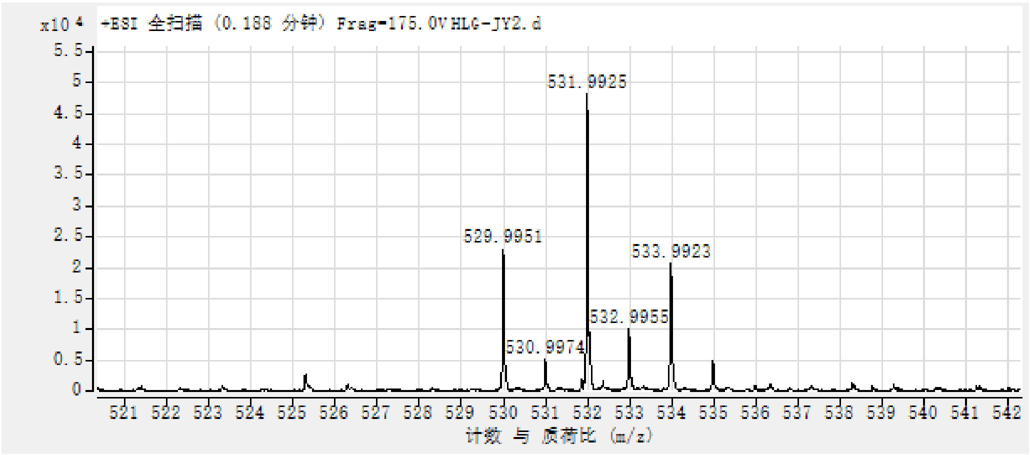

Supplementary Fig. 3 Electrospray ionization mass spectrum of compound **2**.

Synthesis of compound 3

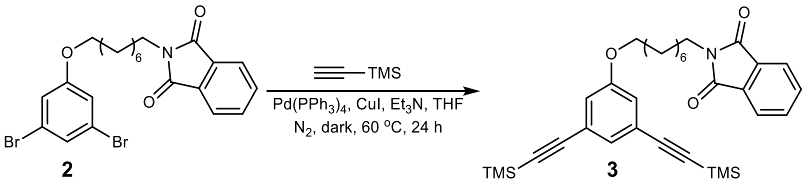

In a 100 mL Schlenk flask were added compound **2** (2.00 g, 3.93 mmol) tetrakis(triphenylphosphine)palladium (230 mg, 0.20 mmol), and cuprous iodide (44 mg, 0.24 mmol). The mixture was degassed with N_2 and the flask was wrapped in aluminum foil to exclude light. Then 30 mL of freshly distilled THF, 12 mL of dry triethylamine and 3 mL of trimethylsilyl acetylene were added to the flask via a syringe under N_2. The mixture was stirred at 60 °C for 24 h. After removal of the solvents, the residue was suspended in ethyl acetate. The solution was washed with water and brine, and then dried over anhydrous Na_2SO_4. The solution was concentrated and the resultant residue was purified by flash column chromatography (ethyl acetate/petroleum ether, 1:100 *v*/*v*) to afford compound **3** as a white solid (1.99 g, 93%). Mp 83.7–84.6 °C. The ^{1}H NMR spectrum of compound **3** is shown in Supplementary Fig. 4. ^{1}H NMR (500 MHz, $CDCl_3$, 298 K) δ 7.86–7.79 (m, 2H), 7.73–7.66 (m, 2H), 7.16 (t, J = 2 Hz, 1H), 6.91 (d, J = 1 Hz, 2H), 3.89 (t, J = 7 Hz, 2H), 3.67 (t, J = 7 Hz, 2H), 1.73 (dd, J = 14, 7 Hz, 2H), 1.68 (dd, J = 12, 5 Hz, 2H), 1.44–1.38 (m, 2H), 1.37–1.32 (m, 6H), 0.24–0.21 (m, 18H). The ^{13}C NMR spectrum of compound **3** is shown in Supplementary Fig. 5. ^{13}C NMR (126 MHz, $CDCl_3$, 298 K) δ 168.6, 158.7, 133.9, 132.3, 128.1, 124.3, 123.3, 118.4, 104.3, 94.6, 68.2, 38.1, 29.3, 29.2, 29.2, 28.7, 26.9, 26.0. HRMS (ESI/Q-TOF) of compound **3** is shown in Supplementary Fig. 6. *m*/*z*: $[M + Na]^+$ calcd for $C_{32}H_{41}NNaO_3Si_2$, 566.2517; found, 566.2523. *m*/*z*: $[M + K]^+$ calcd for $C_{32}H_{41}NKO_3Si_2$, 582,2257; found, 582.2260.

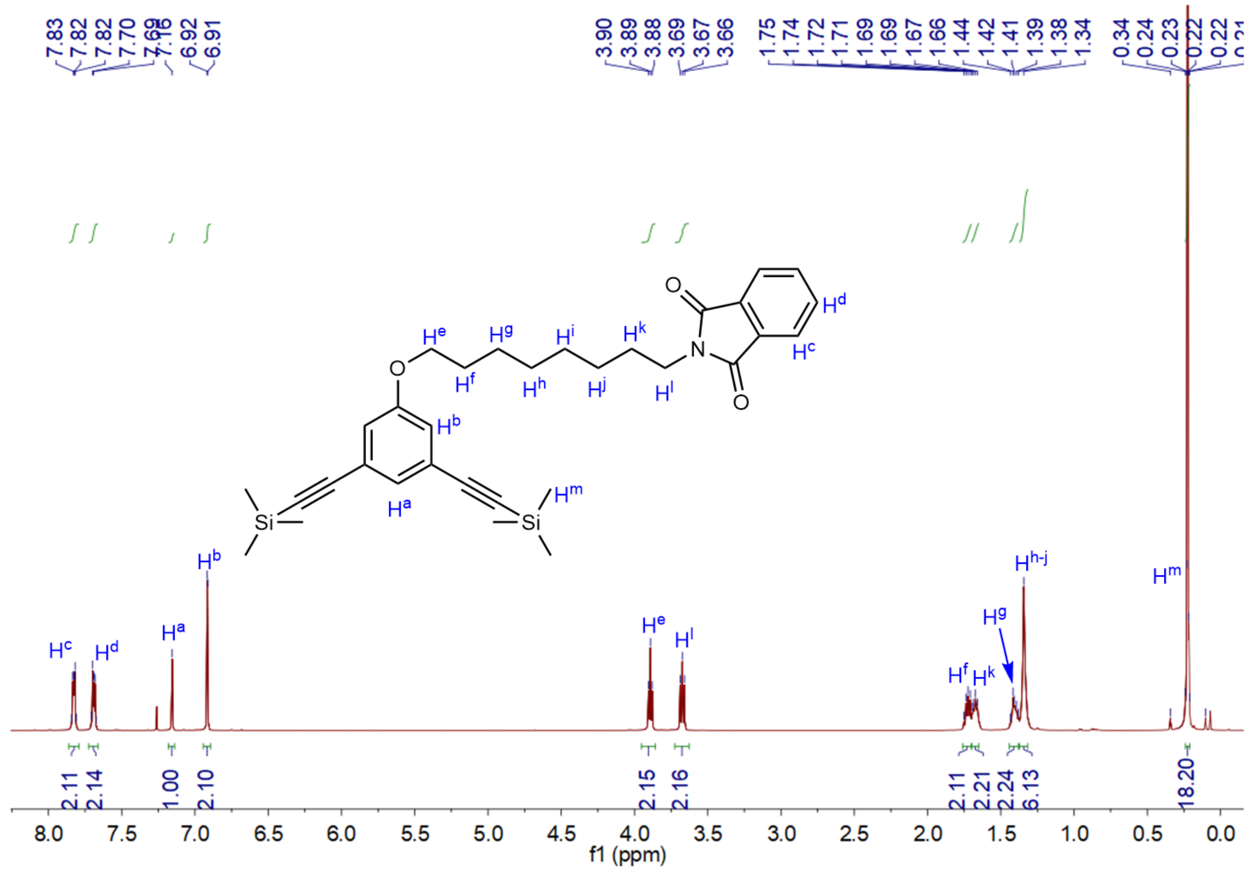

Supplementary Fig. 4 ^{1}H NMR spectrum (500 MHz, $CDCl_3$, 298 K) of compound **3**.

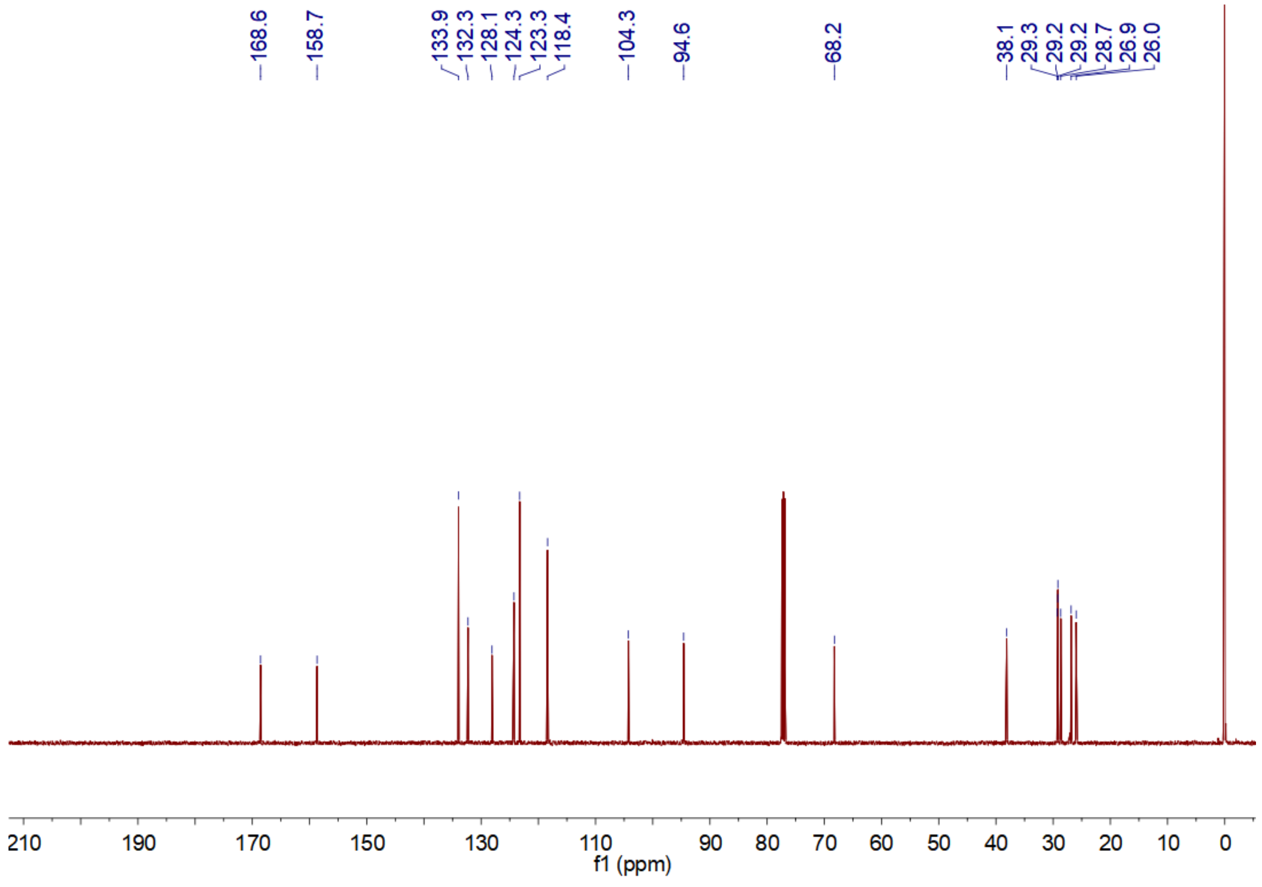

Supplementary Fig. 5 ^{13}C NMR spectrum (126 MHz, $CDCl_3$, 298 K) of compound **3**.

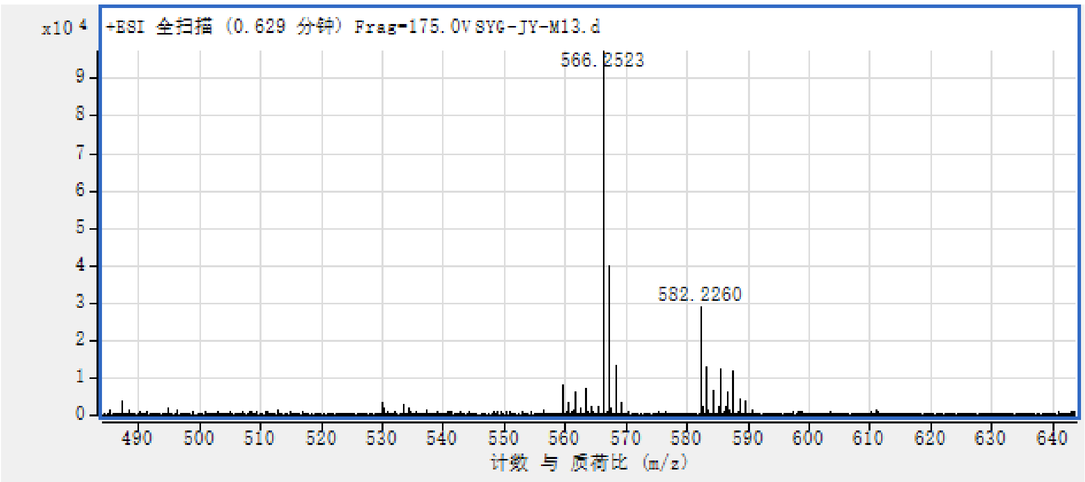

Supplementary Fig. 6 Electrospray ionization mass spectrum of compound **3**.

Synthesis of compound 4

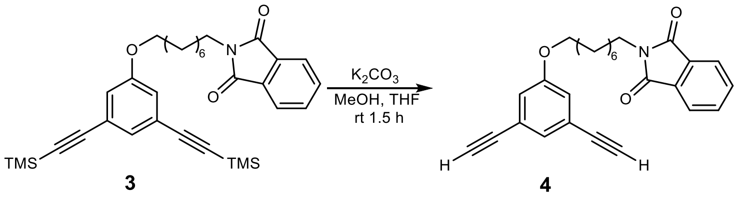

In a 100 mL round-bottomed flask, compound **3** (2.99 g, 3.66 mmol), potassium carbonate (0.50 g, 3.62 mmol), 10 mL THF and 40 mL methanol were added. After the mixture was stirred at room temperature for 30 min, the solvents were removed under vacuum and the residue was suspended in ethyl acetate. The solution was washed with water and brine, and dried over anhydrous Na_2SO_4. The solution was concentrated and the resulting residue was purified by flash column chromatography (ethyl acetate/petroleum ether, 1:50 *v*/*v*) to afford compound **4** as a white solid (1.41 g, 96%). Mp 94.2–94.9 °C. The ^{1}H NMR spectrum of compound **4** is shown in Supplementary Fig. **7**. ^{1}H NMR (500 MHz, $CDCl_3$, 298 K) δ 7.84–7.82 (m, 2H), 7.71–7.69 (m, 2H), 7.18 (t, J = 1 Hz, 1H), 6.98 (d, J = 1 Hz, 2H), 3.91 (t, J = 7 Hz, 2H), 3.68 (t, J = 7 Hz, 2H), 3.05 (s, 2H), 1.77–1.71 (m, 2H), 1.70–1.65 (m, 2H), 1.45–1.39 (m, 2H), 1.37–1.33 (m, 6H). The ^{13}C NMR spectrum of compound **4** is shown in Supplementary Fig. 8. ^{13}C NMR (126 MHz, $CDCl_3$, 298 K) δ 168.6, 158.8, 134.0, 132.3, 128.2, 123.4, 123.3, 119.0, 82.8, 77.7, 68.3, 38.1, 29.3, 29.2, 29.1, 28.7, 26.9, 26.0. HRMS (ESI/Q-TOF) of compound **4** is shown in Supplementary Fig. 9. *m*/*z*: $[M + Na]^+$ calcd for $C_{26}H_{25}NNaO_3$, 422.1727; found, 422.1738.

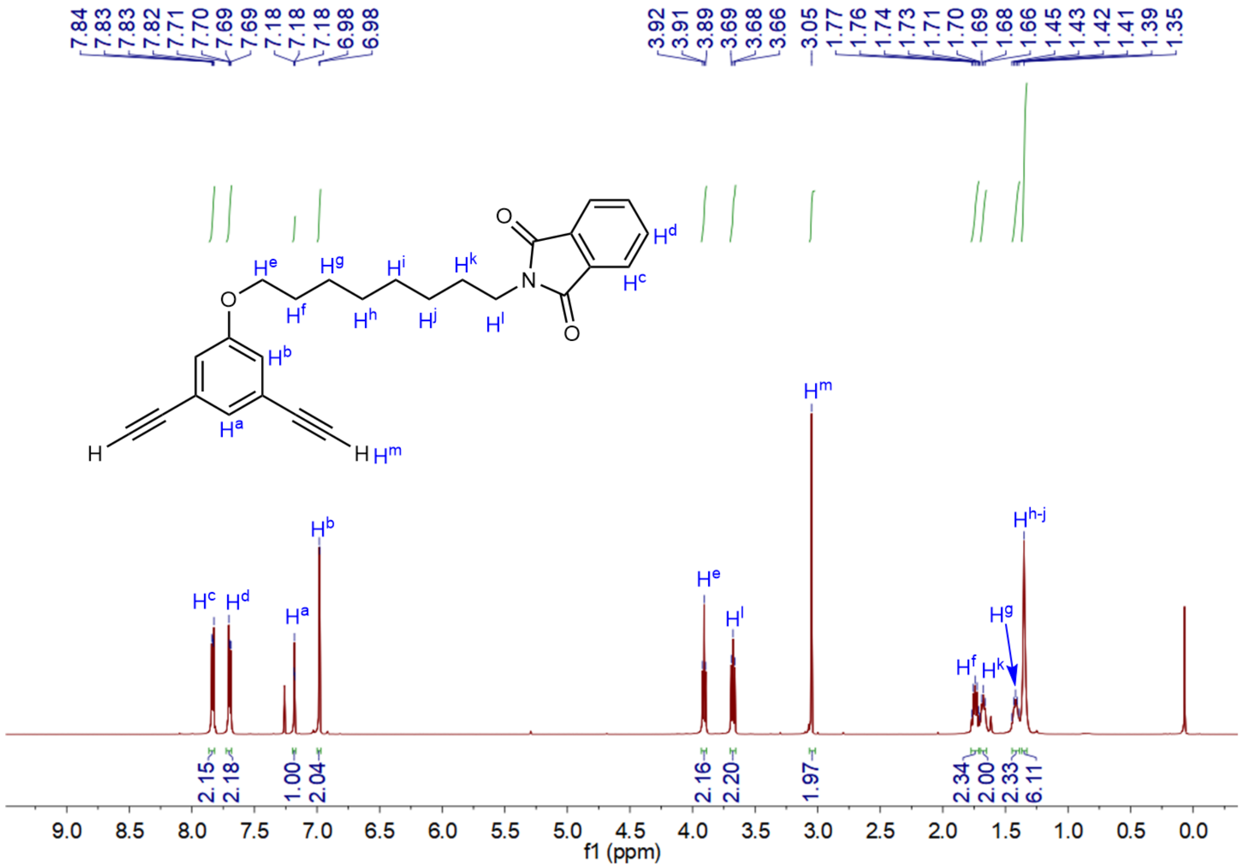

Supplementary Fig. 7 ^{1}H NMR spectrum (500 MHz, $CDCl_3$, 298 K) of compound **4**.

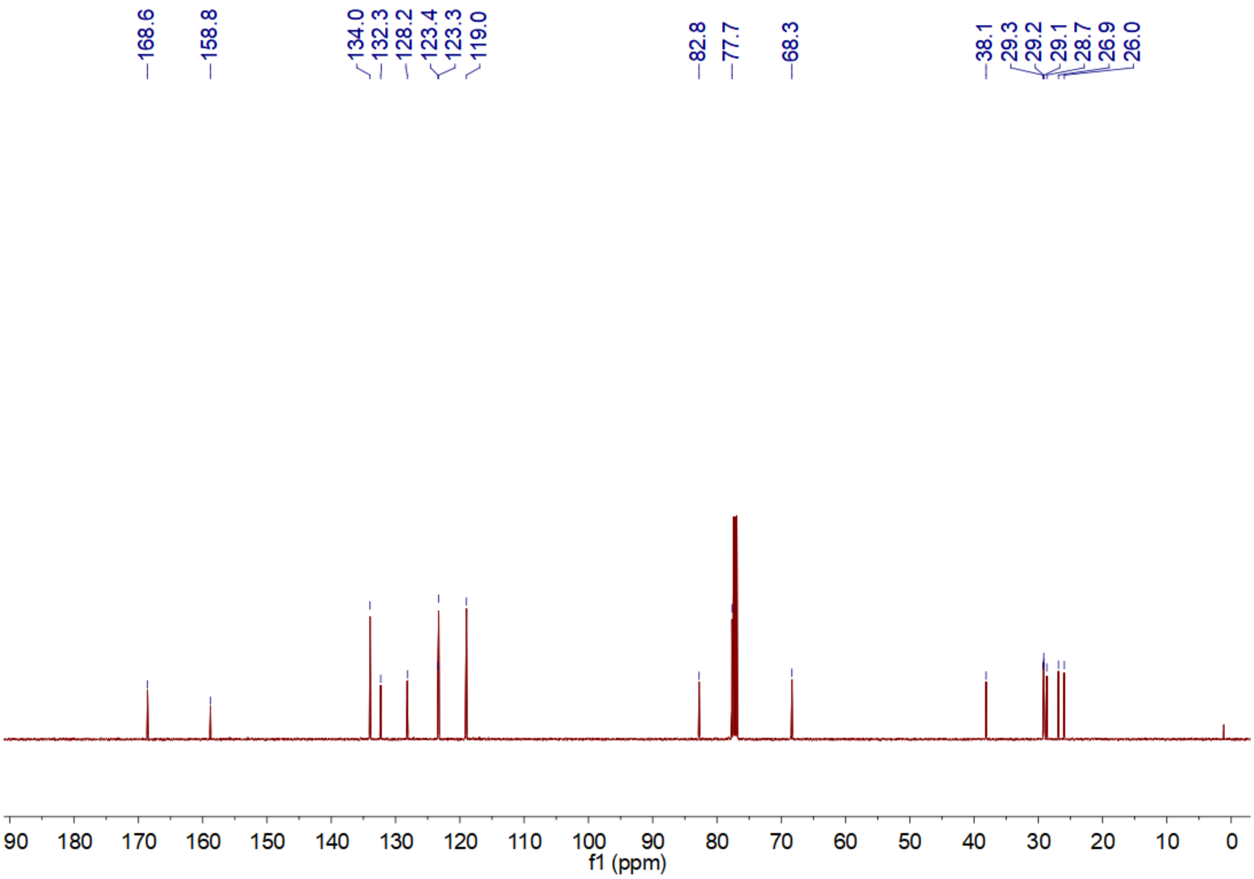

Supplementary Fig. 8 ^{13}C NMR spectrum (126 MHz, $CDCl_3$, 298 K) of compound **4**.

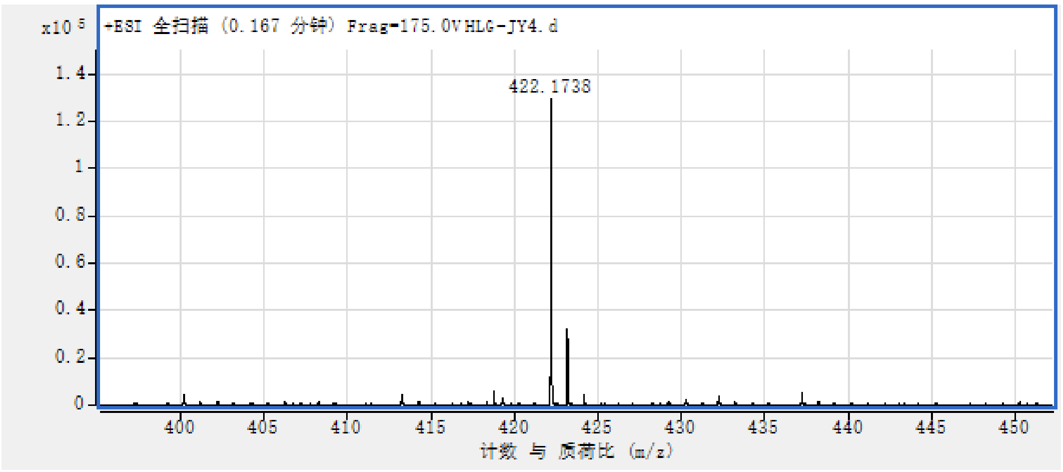

Supplementary Fig. 9 Electrospray ionization mass spectrum of compound **4**.

Synthesis of compound 5

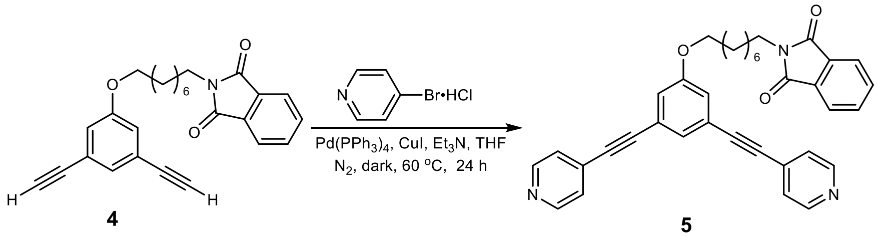

In a 100 mL Schlenk flask, compound **4** (0.80 g, 2.00 mmol), 4-bromopyridine hydrochloride (1.94 g, 10.00 mmol), tetrakis(triphenylphosphine)palladium (120 mg, 0.10 mmol), and cuprous iodide (20 mg, 0.12 mmol) were added. The mixture was degassed with N_2 and the flask was wrapped in aluminum foil to exclude light. Then 40 mL of freshly distilled THF and 8 mL of dry triethylamine were added to the flask via a syringe under N_2. The mixture was stirred at 60 °C for 24 h. After removal of the solvent, the residue was suspended in ethyl acetate and washed with water and brine, and dried over anhydrous Na_2SO_4. The solution was concentrated and the resulting residue was then purified by flash column chromatography (dichloromethane/methanol, 150:1 *v*/*v*) to afford compound **5** as a white solid (0.93 g, 76%). Mp 114.3–114.8 °C. The ^{1}H NMR spectrum of compound **5** is shown in Supplementary Fig. 10. ^{1}H NMR (500 MHz, $CDCl_3$, 298 K) δ 8.61 (dd, J = 5, 1 Hz, 4H), 7.84–7.82 (m, 2H), 7.70–7.68 (m, 2H), 7.37 (dd, J = 5, 2 Hz, 4H), 7.33 (t, J = 1 Hz, 1H), 7.08 (d, J = 1 Hz, 2H), 3.98 (t, J = 6 Hz, 2H), 3.68 (t, J = 7 Hz, 2H), 1.83–1.75 (m, 2H), 1.73–1.65 (m, 2H), 1.49–1.42 (m, 2H), 1.40–1.34 (m, 6H). The ^{13}C NMR spectrum of compound **5** is shown in Supplementary Fig. 11. ^{13}C NMR (126 MHz, $CDCl_3$, 298 K) δ 168.6,

159.1, 149.9, 134.0, 132.3, 131.2, 127.6, 125.7, 123.6, 123.3, 118.9, 92.9, 87.2, 68.5, 38.1, 29.2, 29.2, 28.7, 26.9, 26.0. HRMS (ESI/Q-TOF) of compound **5** is shown in Supplementary Fig. 12. *m*/*z*: $[M + H]^+$ calcd for $C_{36}H_{32}N_3O_3$, 554.2438; found, 554.2477.

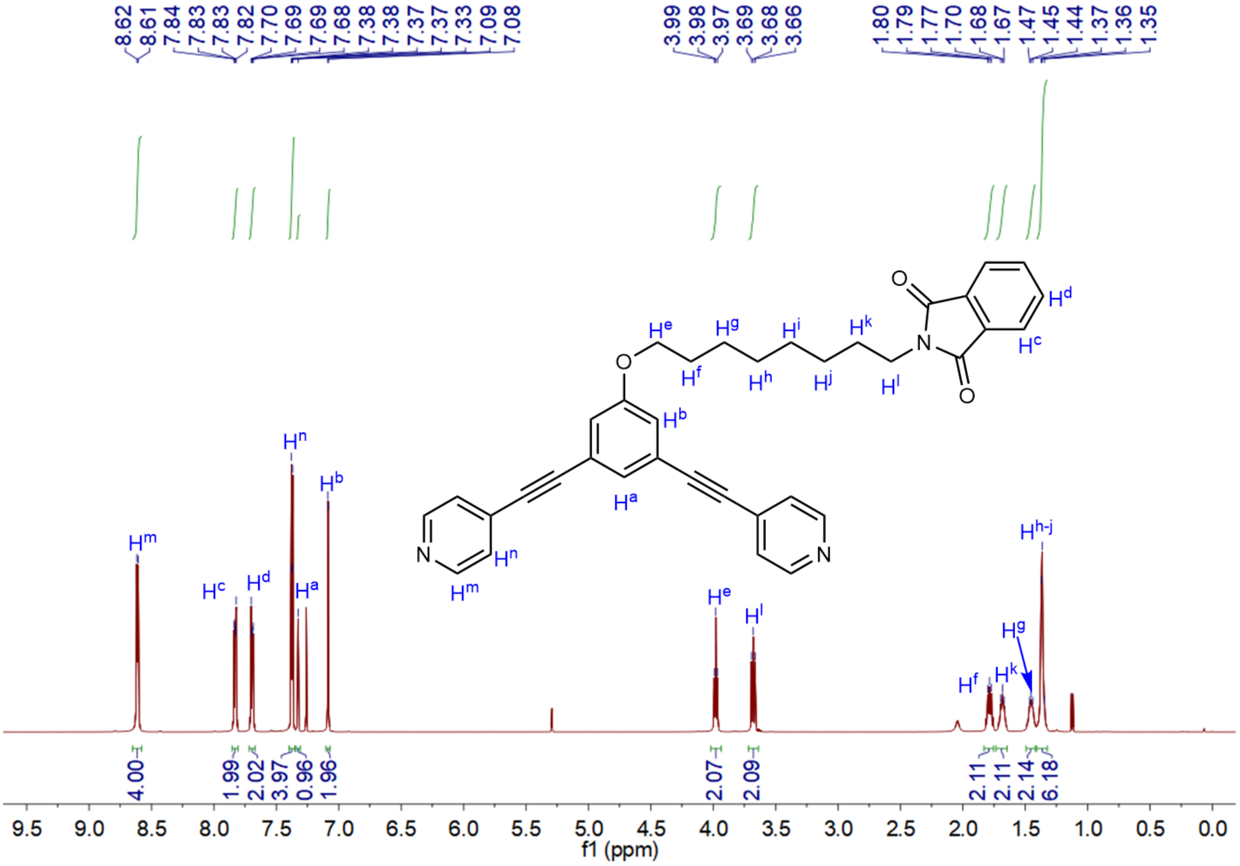

Supplementary Fig. 10 ^{1}H NMR spectrum (500 MHz, $CDCl_3$, 298 K) of compound **5**.

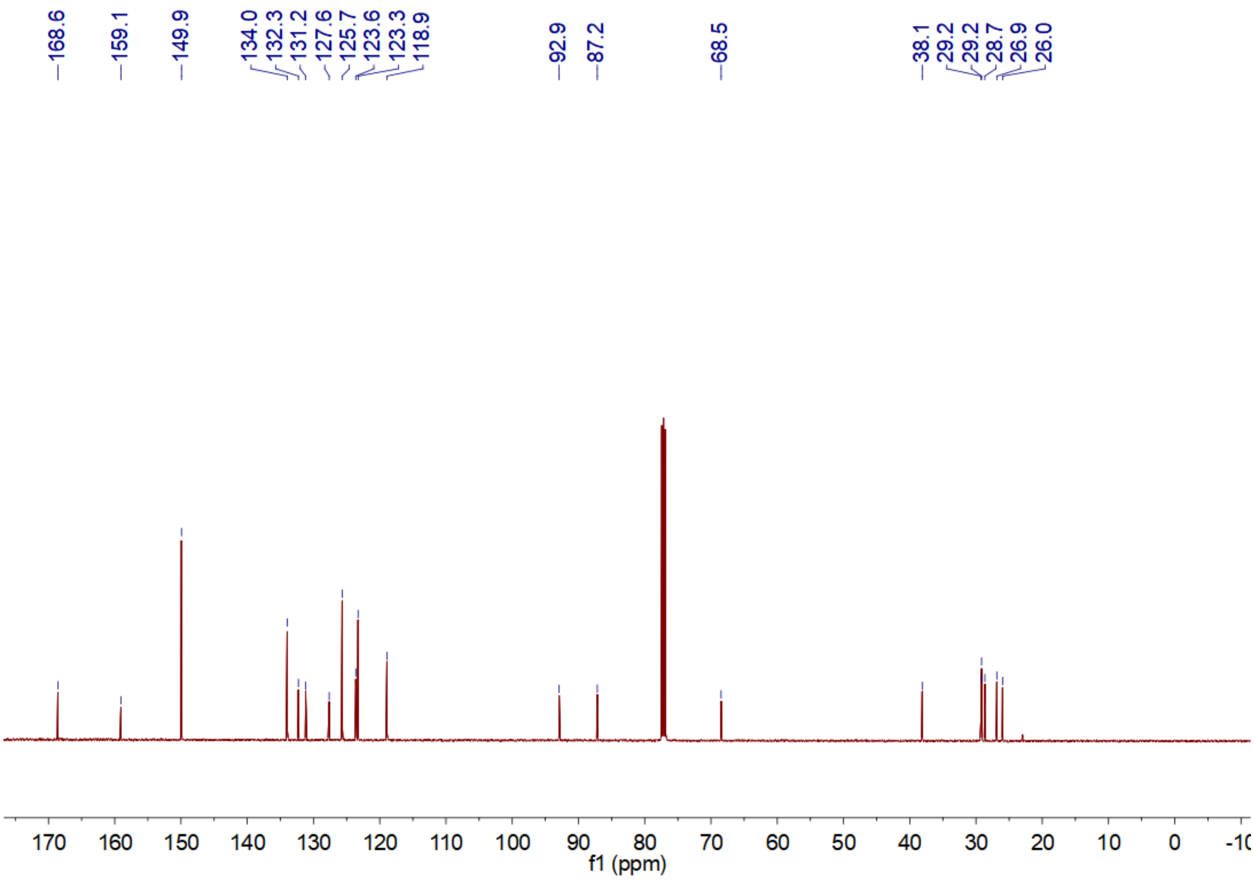

Supplementary Fig. 11 ^{13}C NMR spectrum (126 MHz, $CDCl_3$, 298 K) of compound **5**.

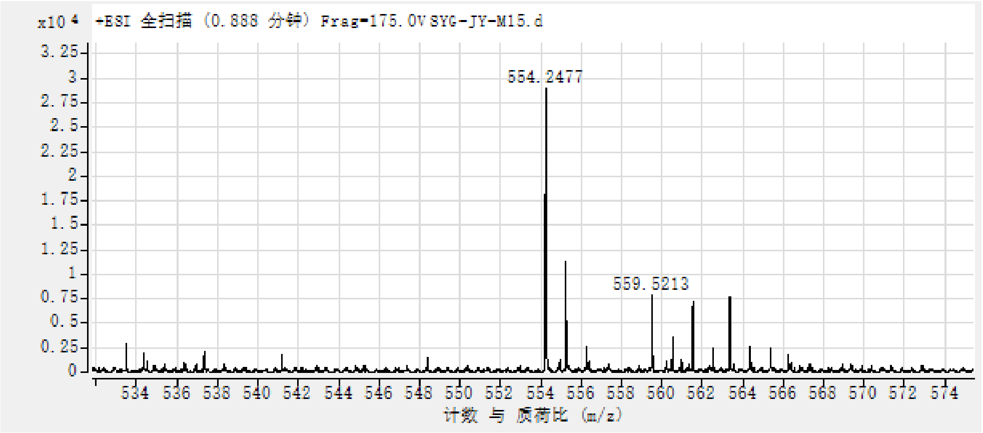

Supplementary Fig. 12 Electrospray ionization mass spectrum of compound **5**.

Synthesis of compound 6

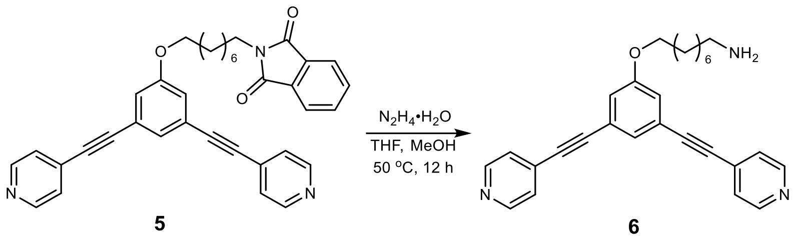

Compound **5** (2.00 g, 3.62 mmol) was dissolved in a mixture of THF (20 mL) and CH_3OH (50 mL). Hydrazine monohydrate (5 mL) was added under N_2 atmosphere. The mixture was then stirred at 50 °C for 12 h. After removal of the solvent, the residue was suspended in $CHCl_3$ and washed with water and brine, and dried over anhydrous Na_2SO_4. The solution was concentrated and the resulted residue was then purified by flash column chromatography (dichloromethane/methanol, 100:1 *v*/*v*) to afford compound **6** as pale yellow oil (1.27 g, 84%). The ^{1}H NMR spectrum of **6** is shown in Supplementary Fig. 13. ^{1}H NMR (500 MHz, $CDCl_3$, 298 K) δ 8.61 (dd, J = 5, 1 Hz, 4H), 7.37 (dd, J = 5, 1 Hz, 4H), 7.33 (t, J = 1 Hz, 1H), 7.09 (d, J = 1 Hz, 2H), 3.99 (t, J = 6 Hz, 2H), 2.70 (t, J = 7 Hz, 2H), 1.85–1.76 (m, 2H), 1.76–1.73 (m, 2H), 1.48–1.44 (m, 4H), 1.40–1.30 (m, 6H). The ^{13}C NMR spectrum of **6** is shown in Supplementary Fig. 14. ^{13}C NMR (101 MHz, $CDCl_3$, 298 K) δ 159.3, 150.1, 131.3 127.9, 125.8, 123.9, 119.1, 93.1, 87.4, 68.7, 42.1, 33.0, 29.6. 29.6, 29.4, 27.1, 26.2. HRMS (ESI/Q-TOF) of compound **6** is shown in Supplementary Fig. 15. *m*/*z*: $[M + H]^+$ calcd for $C_{28}H_{30}N_3O$, 424.2383; found, 424.2393.

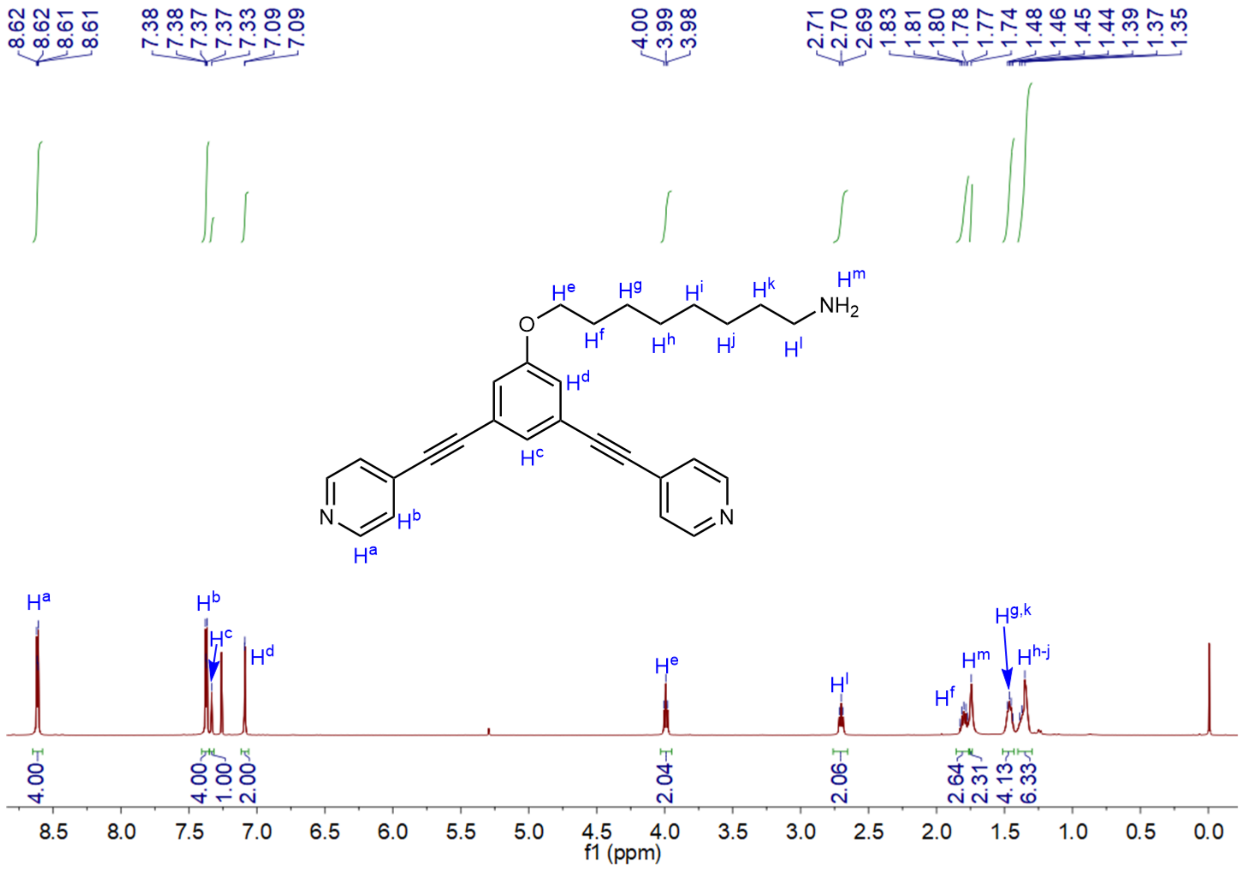

Supplementary Fig. 13 ^{1}H NMR spectrum (500 MHz, $CDCl_3$, 298 K) of compound **6**.

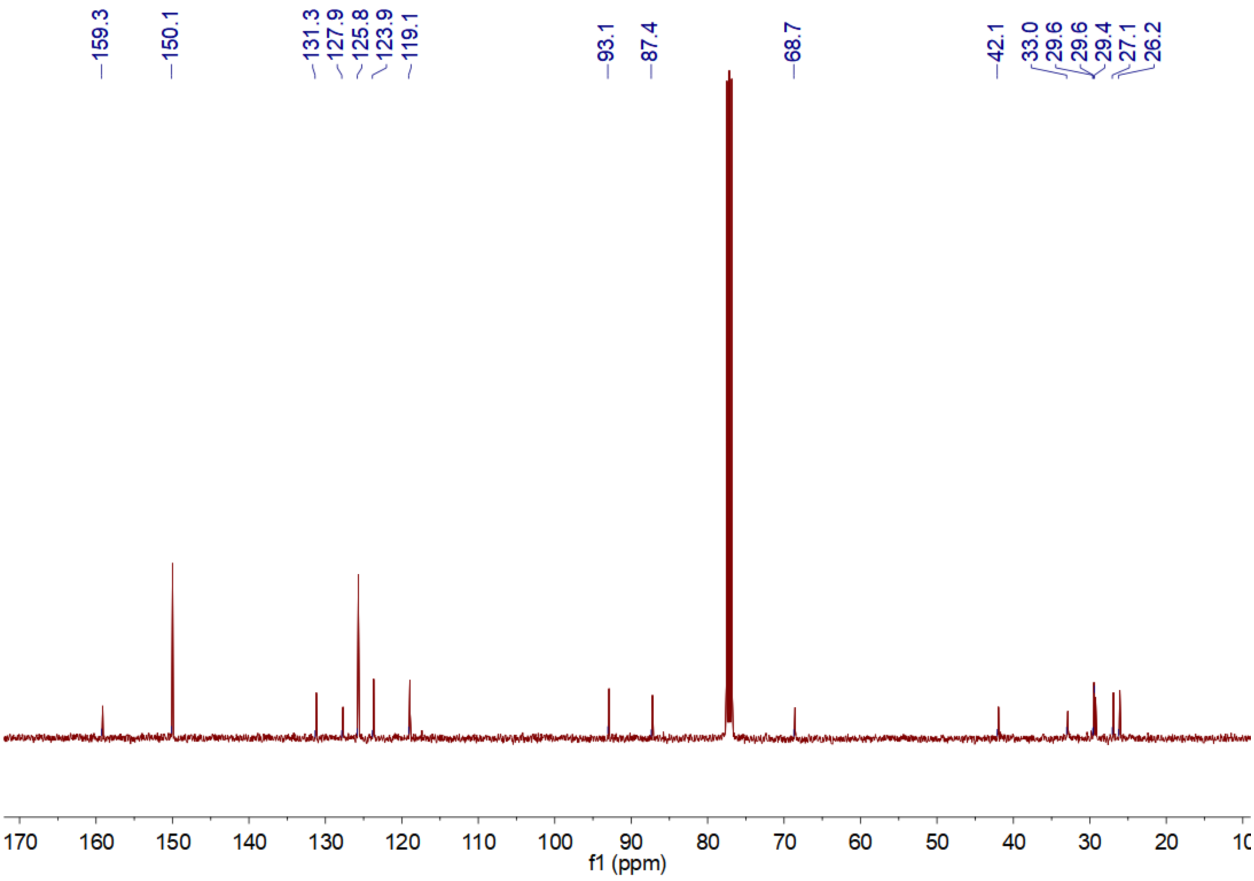

Supplementary Fig. 14 ^{13}C NMR spectrum (126 MHz, $CDCl_3$, 298 K) of compound **6**.

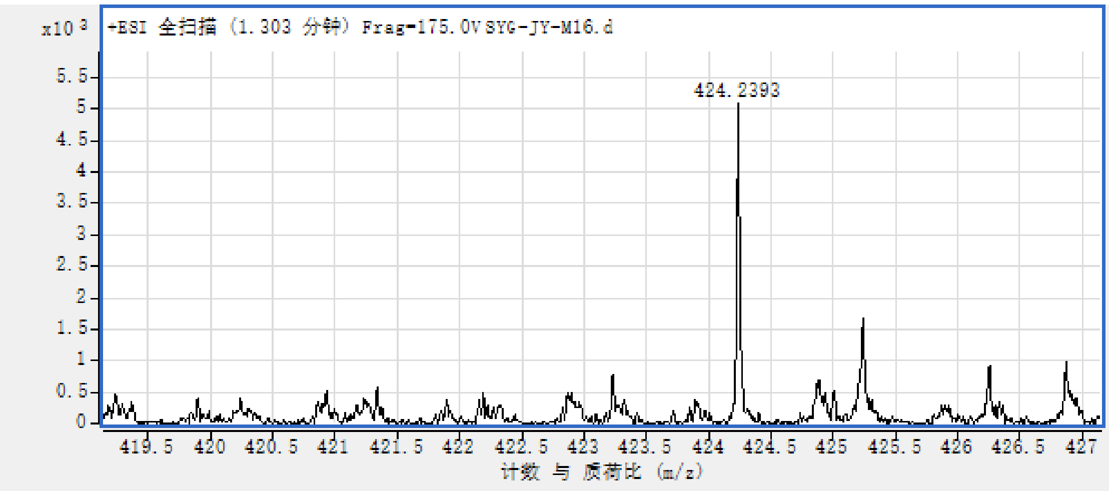

Supplementary Fig. 15 Electrospray ionization mass spectrum of compound **6**.

Synthesis of monomer M-1

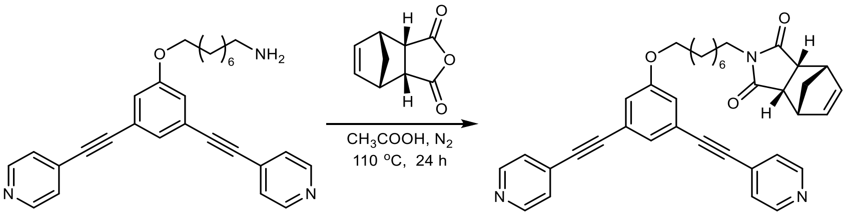

6 **M-1**

A mixture of compound **6** (0.50 g, 1.18 mmol), *endo*-5-norbornene-2,3-dicarboxylic anhydride (0.78 g, 4.72 mmol) and glacial acetic acid (50 mL) was stirred for 12 h at reflux under N_2 atmosphere. After the solvent was removed under vacuum, CH_2Cl_2 (100 mL) was added to dissolve the residue. The solution was washed with water and brine, and then dried over anhydrous Na_2SO_4. After removal of the solvent, the residue was purified by flash column chromatography (ethyl acetate/petroleum ether, 1:1 *v*/*v*) to afford **M-1** as a white solid (0.54 g, 80 %). Mp 127.6–128.3 °C. The ^{1}H NMR spectrum of **M-1** is shown in Supplementary Fig. 16. ^{1}H NMR (500 MHz, CD_2Cl_2, 298 K) δ 8.60 (dd, J = 4, 2 Hz, 4H), 7.40 (dd, J = 4, 2 Hz, 4H), 7.34 (t, J = 1 Hz, 1H), 7.12 (d, J = 1 Hz, 2H), 6.07 (t, J = 2 Hz, 2H), 4.00 (t, J = 7 Hz, 2H), 3.33–3.32 (m, 2H), 3.28 (t, J = 7 Hz, 2H), 3.21–3.20 (m, 2H), 1.83–1.76 (m, 2H), 1.69 (dt, J = 9, 2 Hz, 1H), 1.52 (dt, J = 9, 2 Hz, 1H), 1.48–1.40 (m, 4H), 1.37–1.30 (m, 4H), 1.27–1.22 (m, 2H). The ^{13}C NMR spectrum of **M-1** is shown in Supplementary Fig. 17. ^{13}C NMR (126 MHz, $CDCl_3$, 298 K) δ 159.1, 150.0, 131.2, 127.7, 125.7, 123.7, 118.9, 92.9, 87.2, 68.6, 42.0, 32.9, 29.5, 29.4, 26.9, 26.1. HRMS (ESI/Q-TOF)

of **M-1** is shown in Supplementary Fig. 18. *m*/*z*: $[M + H]^+$ calcd for $C_{37}H_{36}N_3O_3$, 570.2751; found, 570.2765.

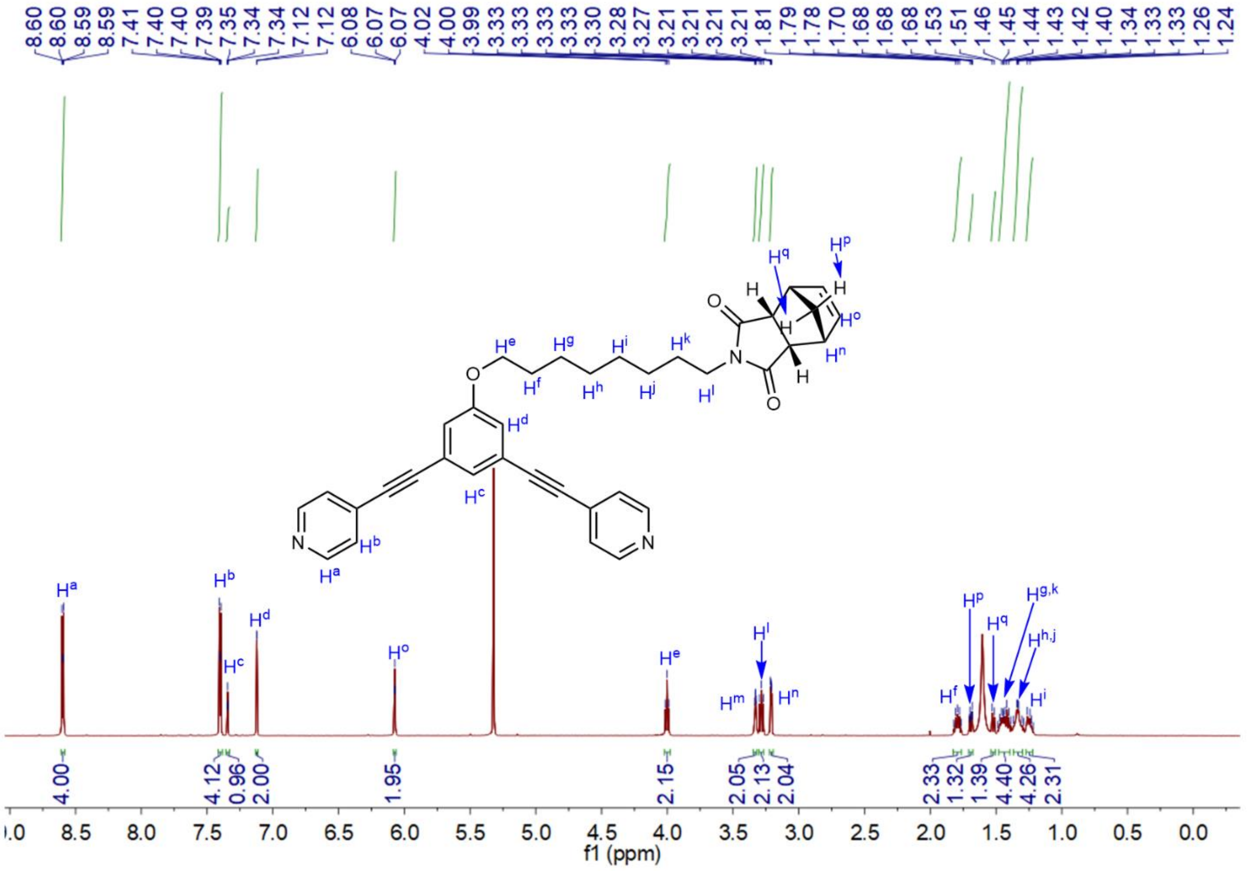

Supplementary Fig. 16 ^{1}H NMR spectrum (500 MHz, CD_2Cl_2, 298 K) of monomer **M-1**.

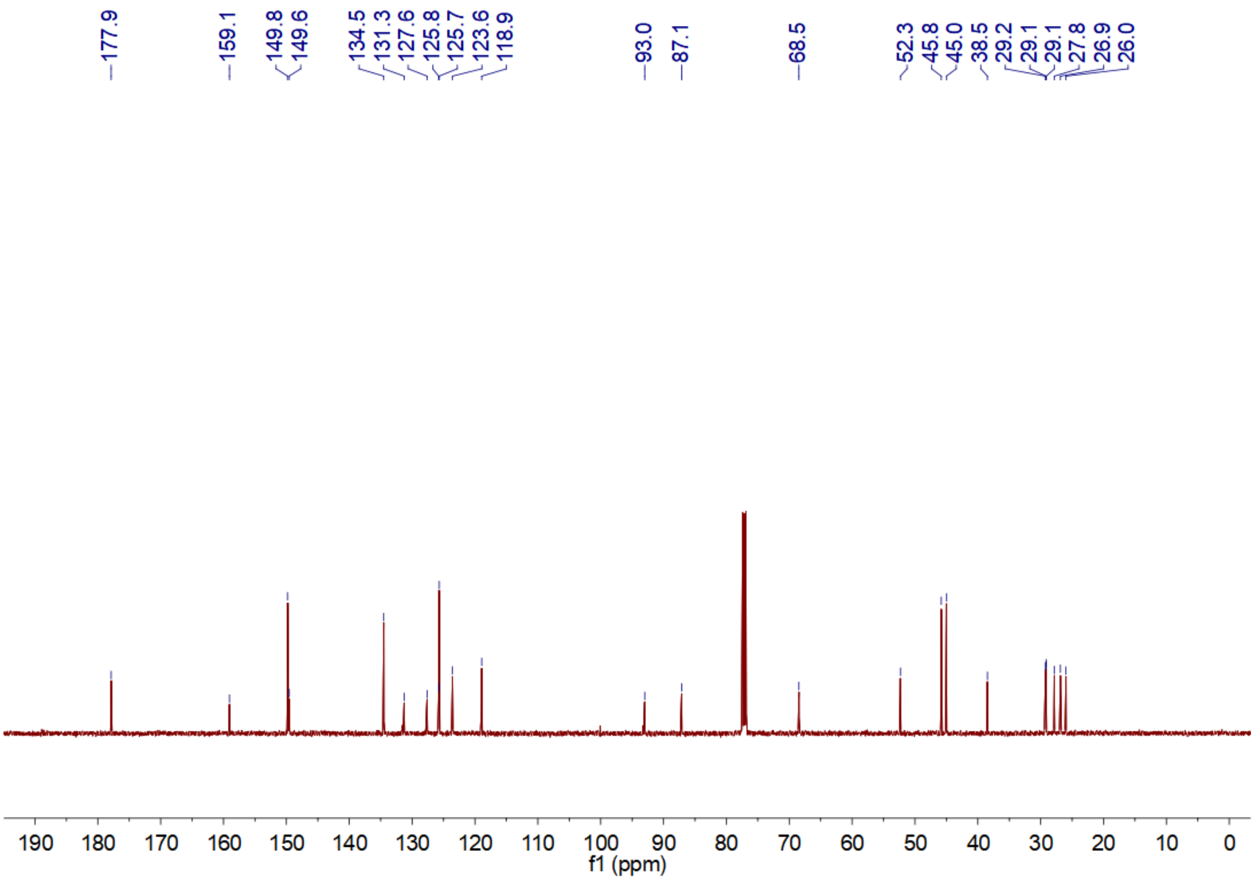

Supplementary Fig. 17 ^{13}C NMR spectrum (126 MHz, $CDCl_3$, 298 K) of monomer **M-1**.

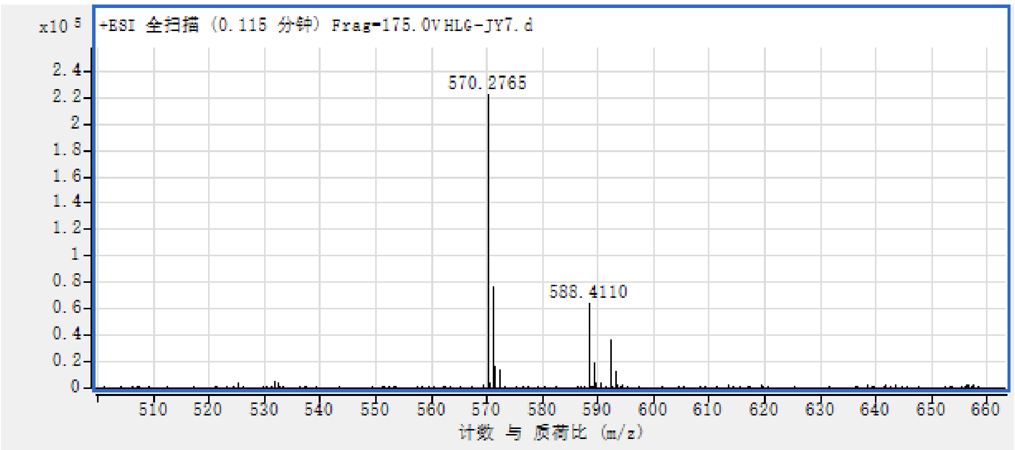

Supplementary Fig. 18 Electrospray ionization mass spectrum of monomer **M-1**.

3. Synthesis of 60° acceptor 7 and 120° acceptor 8

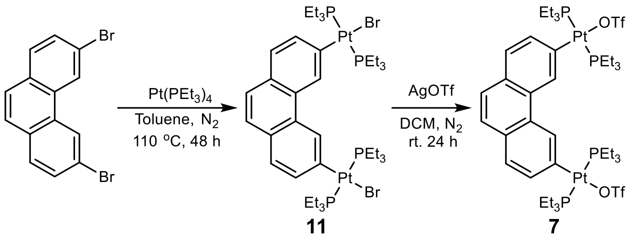

A 50 mL Schlenk flask wrapped in aluminum foil was charged with 2,9-dibromophenanthrene (400 mg, 1.19 mmol) and $Pt(PEt_3)_4$ (1990 mg, 3 mmol) under N_2 atmosphere. Freshly distilled toluene (40 mL) was added to the flask under nitrogen atmosphere with a syringe, and the resulting solution was stirred for 24 h at 45 °C. After removal of the solvent, the residue was purified by flash column chromatography (dichloromethane/hexane, 1:10 *v*/*v*) to afford compound **11** as a white solid (875 mg, 61 %). ^{1}H NMR (500 MHz, $CDCl_3$, 298 K) δ 8.56 (s, 2H), 7.57 (d, J = 8 Hz, 2H), 7.49 (s, 2H), 7.42 (d, J = 8 Hz, 2H), 1.68 (ddt, J = 11, 7, 4 Hz, 24H), 1.07 (m, 36H). ^{31}P{^{1}H} NMR (202 MHz, $CDCl_3$, 298 K) δ 12.24 (s, ^{195}Pt satellites, $J_{Pt\text{-}P}$ = 2760 Hz).

Compound **11** (200 mg, 0.17 mmol) and AgOTf (128 mg, 0.50 mmol) were placed in a 30 mL Schlenk flask followed by adding 10 mL of dichloromethane. The reaction was stirred in the dark at room temperature for 24 h. A clear solution with a heavy creamy precipitate resulted, the precipitate was filtered off and the solvent was removed under a flow of N_2. The residue was redissolved in a minimal amount of dichloromethane, and then *n*-pentane was carefully added to precipitate the residual AgOTf. The cloudy solution that resulted was filtered through a glass fiber filter, and the product was then precipitated by adding more *n*-pentane. The supernatant was decanted, and the product was dried in vacuo overnight to afford compound **7** as a white solid (214 mg, 96%). The ^{1}H NMR spectrum of **7** is shown in Supplementary Fig. 19. ^{1}H NMR (500 MHz, CD_2Cl_2, 298 K) δ 8.46 (s, 2H), 7.60 (d, J = 8 Hz, 2H), 7.44 (d, J = 8 Hz, 2H), 1.67 (m, 24H), 1.21–1.14 (m, 36H). The ^{31}P NMR spectrum of **7** is shown in Supplementary Fig. 20. ^{31}P{^{1}H} NMR (202 MHz, CD_2Cl_2, 298 K) δ 19.54 (s, ^{195}Pt satellites, $J_{Pt\text{-}P}$ = 2830 Hz).

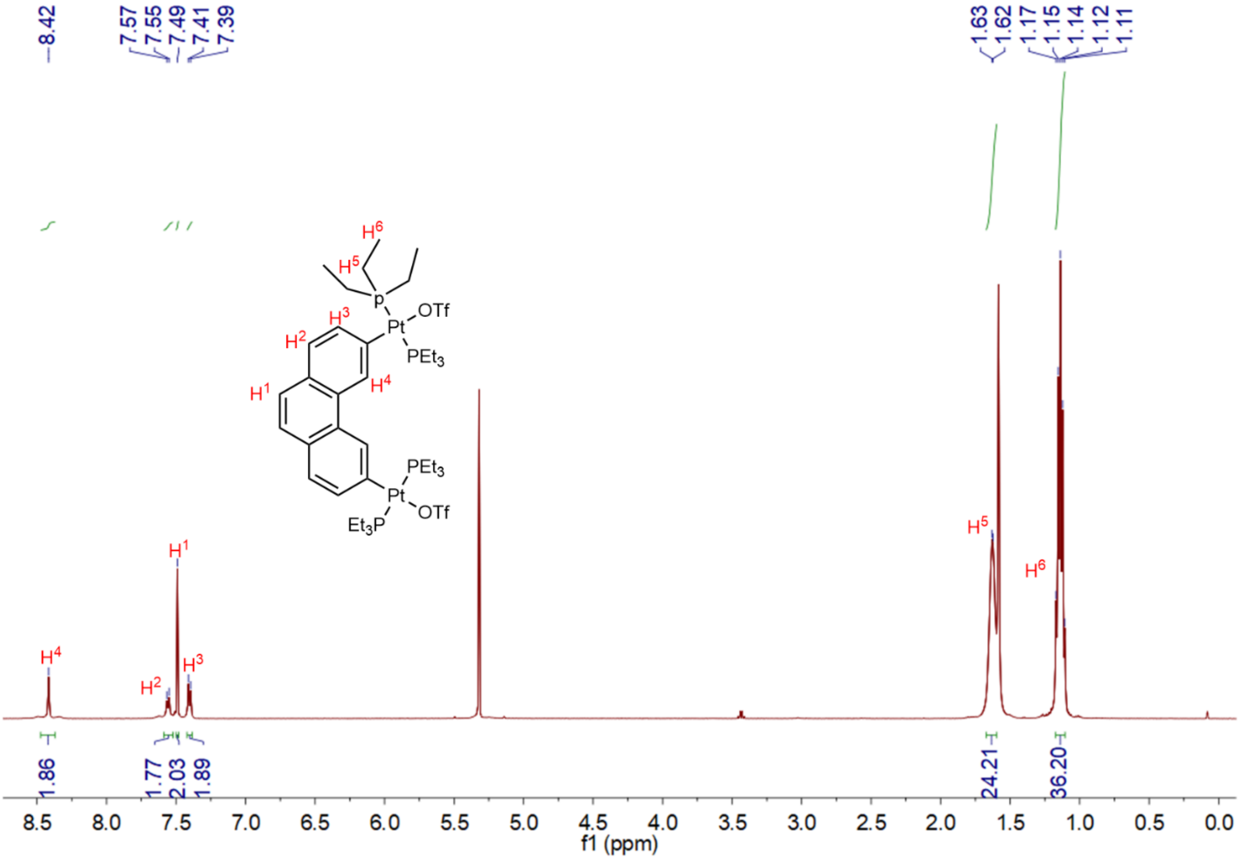

Supplementary Fig. 19 ^{1}H NMR spectrum (500 MHz, CD_2Cl_2, 298 K) of acceptor **7**.

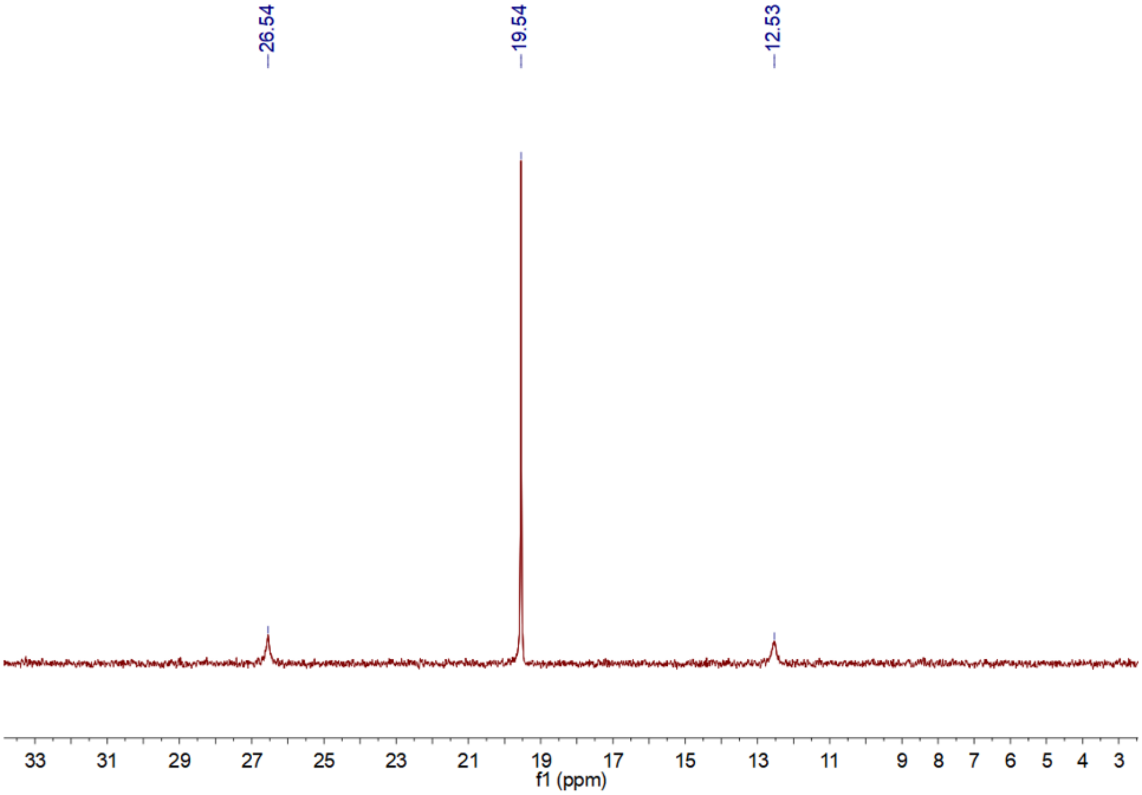

Supplementary Fig. 20 $^{31}P\{^{1}H\}$ NMR spectrum (202 MHz , CD_2Cl_2, 298 K) of acceptor **7**.

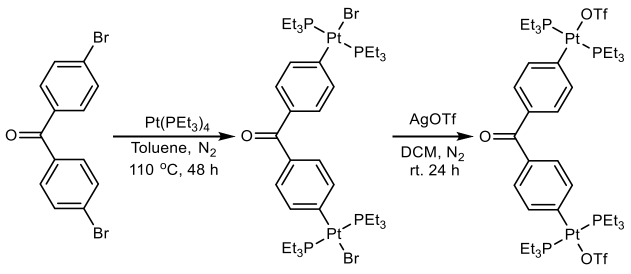

12 **8**

Compound **12** was synthesized by using a method similar to that for **11**. ^{1}H NMR (500 MHz, $CDCl_3$, 298 K) δ 7.51 (d, J = 8 Hz, 4H), 7.39 (d, J = 8 Hz, 4H), 1.76–1.68 (m, 24H), 1.12–1.05 (m, 36H). ^{31}P{^{1}H} NMR (202 MHz, $CDCl_3$, 298 K) δ 11.92 (s, ^{195}Pt satellites, $J_{Pt\text{-}P}$ = 2720 Hz).

Compound **8** was synthesized by using a method similar to that for **7**. The ^{1}H NMR spectrum of **8** is shown in Supplementary Fig. 21. ^{1}H NMR (500 MHz, CD_2Cl_2, 298 K) δ 7.45 (d, J = 8 Hz, 4H), 7.31 (d, J = 8 Hz, 4H), 1.65 (m, 24H), 1.17–1.11 (m, 36H). The ^{31}P NMR spectrum of **7** is shown in Supplementary Fig. 22. ^{31}P{^{1}H} NMR (202 MHz, CD_2Cl_2, 298 K) δ 19.23 (s, ^{195}Pt satellites, $J_{Pt\text{-}P}$ = 2782 Hz).

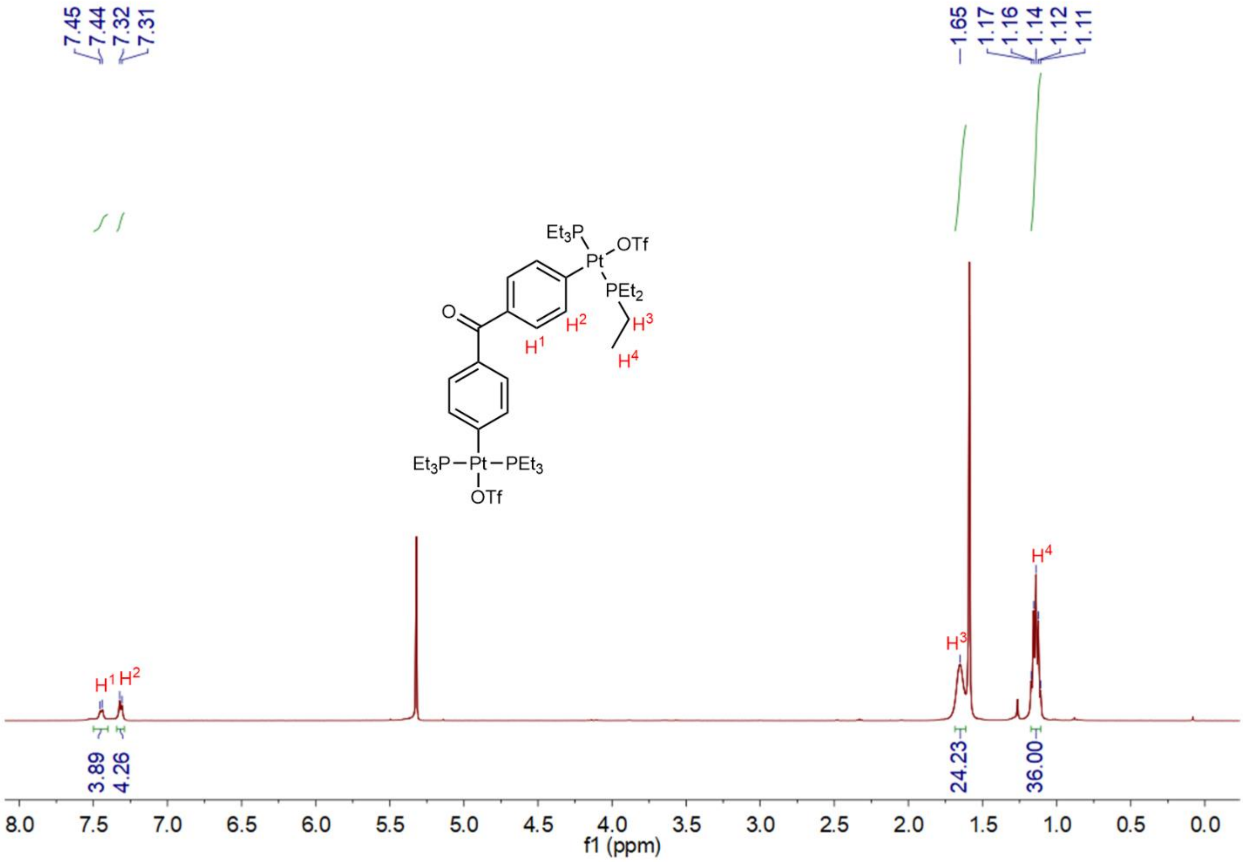

Supplementary Fig. 21 ^{31}P {^{1}H} NMR spectrum (202 MHz, CD_2Cl_2, 298 K) of acceptor **8**.

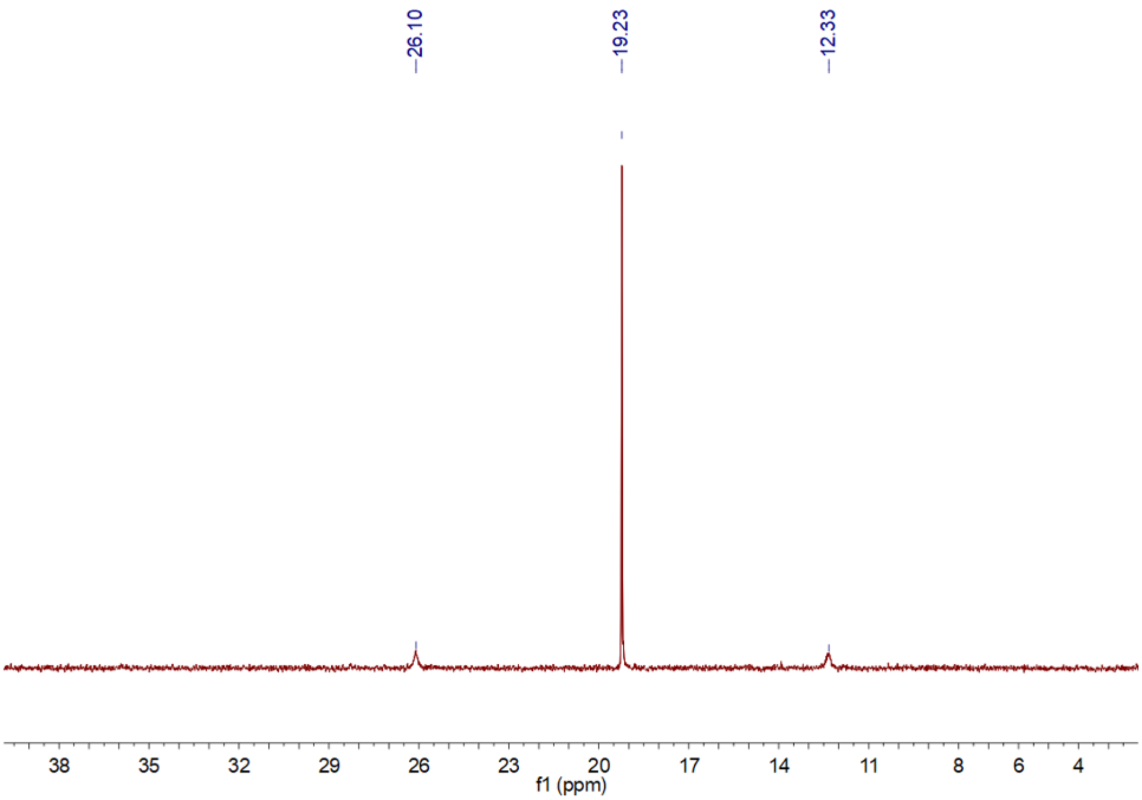

Supplementary Fig. 22 $^{31}P\{^1H\}$ NMR spectrum (202 MHz, CD_2Cl_2, 298 K) of acceptor **8**.

4. Synthesis of norbornene-functionalized rhomboid 9

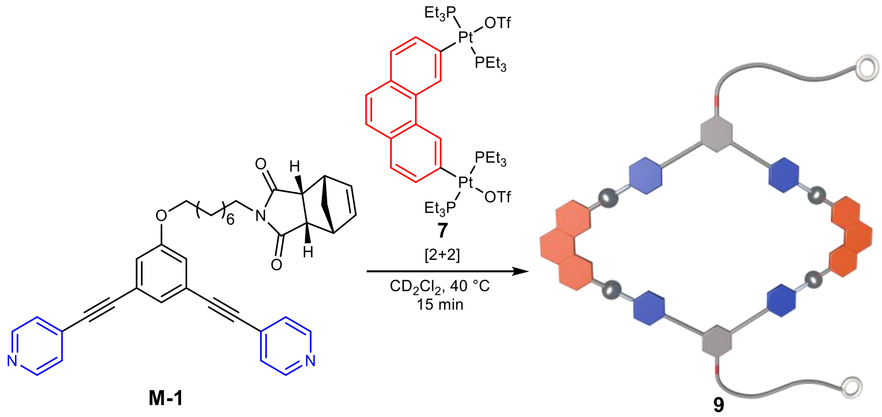

Monomer **M-1** (3.42 mg, 0.006 mmol) and 60° diplatinum acceptor **7** (8.02 mg, 0.006 mmol) were dissolved in 0.6 mL of CD_2Cl_2 in a vial. The reaction mixture was heated at 40 °C for 15 min to give a homogeneous solution of metallacycle **9** that was directly used for analysis. The ^1H NMR spectrum of metallacycle **9** is shown in Supplementary Fig. 23. ^1H NMR (500 MHz, CD_2Cl_2, 298 K) δ 9.24 (d, J = 5 Hz, 4H), 8.77 (s, 4H), 8.66 (d, J = 6 Hz, 4H), 7.91 (d, J = 5 Hz, 4H), 7.76 (dd, J = 6, 1 Hz, 4H), 7.60 – 7.59 (m, 14H), 7.29 (d, J = 1 Hz, 4H), 6.09 (t, J = 2 Hz, 4H), 4.07 (t, J = 6 Hz, 4H), 3.35–3.33 (m, 4H), 3.30 (t, J = 7 Hz, 4H), 3.25–3.20 (m, 4H), 1.87–1.81 (m, 4H), 1.70 (dt, J = 9 Hz, 2H), 1.55–1.53 (m, 2H),

1.52–1.44 (m, 8H), 1.41–1.35 (m, 48H), 1.35–1.32 (m, 8H), 1.30–1.24 (m, 4H), 1.18–1.11 (m, 72H). The $^{31}P\{^1H\}$ NMR spectrum of metallacycle **9** is shown in Supplementary Fig. 24. $^{31}P\{^1H\}$ NMR (202 MHz, CD_2Cl_2, 298 K) δ 12.20 (s, ^{195}Pt satellites, $J_{Pt\text{-}P}$ = 2698 MHz). HRMS (ESI/Q-TOF) of **9** is shown in Supplementary Fig. 25. *m*/*z*: $[M - 4OTf]^{4+}$ calcd for $C_{150}H_{206}N_6O_6P_8Pt_4$, 804.3126; found, 804.3121. *m*/*z*: $[M - 3OTf]^{3+}$ calcd for $C_{151}H_{206}F_3N_6O_9P_8Pt_4S$, 1122.0676; found, 1122.0660. *m*/*z*: $[M - 2OTf]^{2+}$ calcd for $C_{152}H_{206}F_6N_6O_{12}P_8Pt_4S_2$, 1757.5775; found, 1757.5877.

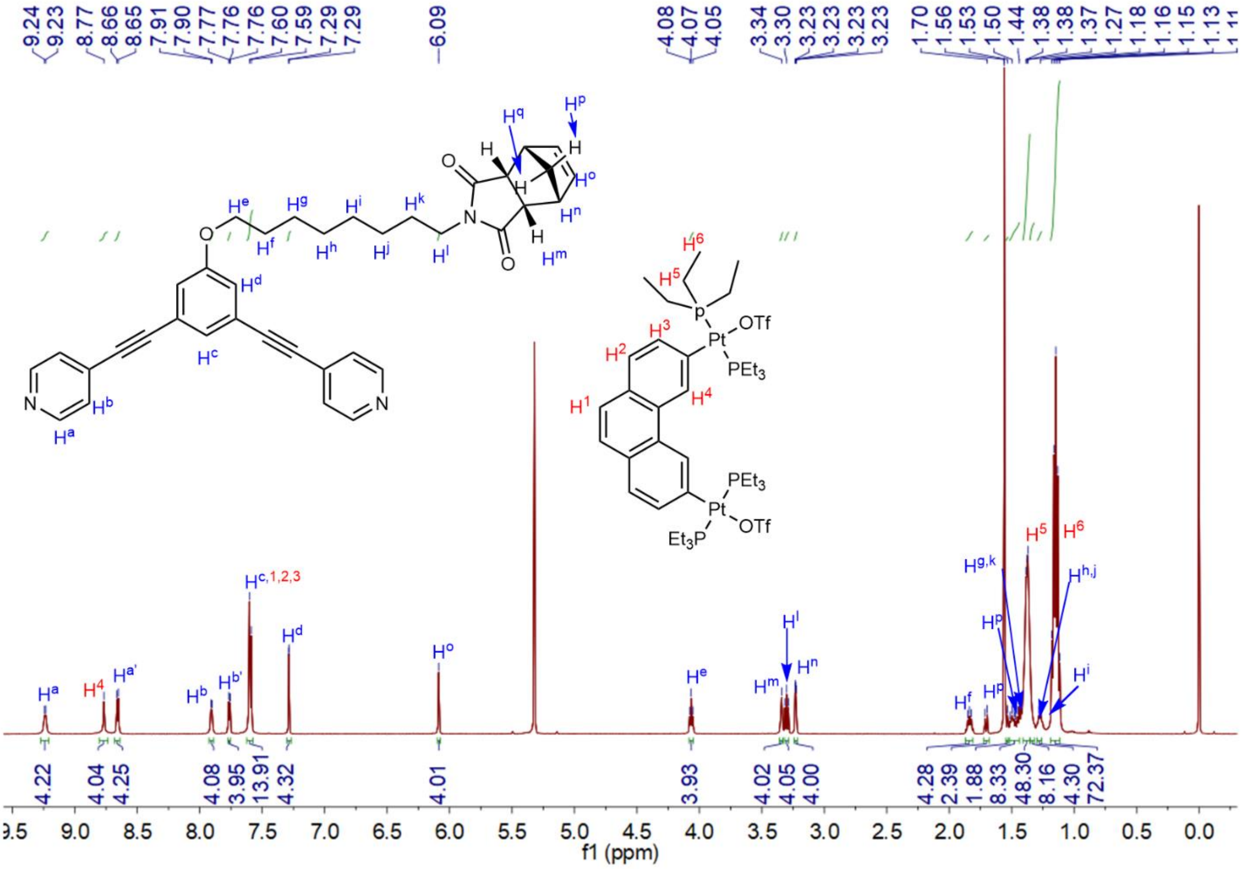

Supplementary Fig. 23 ^1H NMR spectrum (500 MHz, CD_2Cl_2, 298 K) of rhomboid **9**.

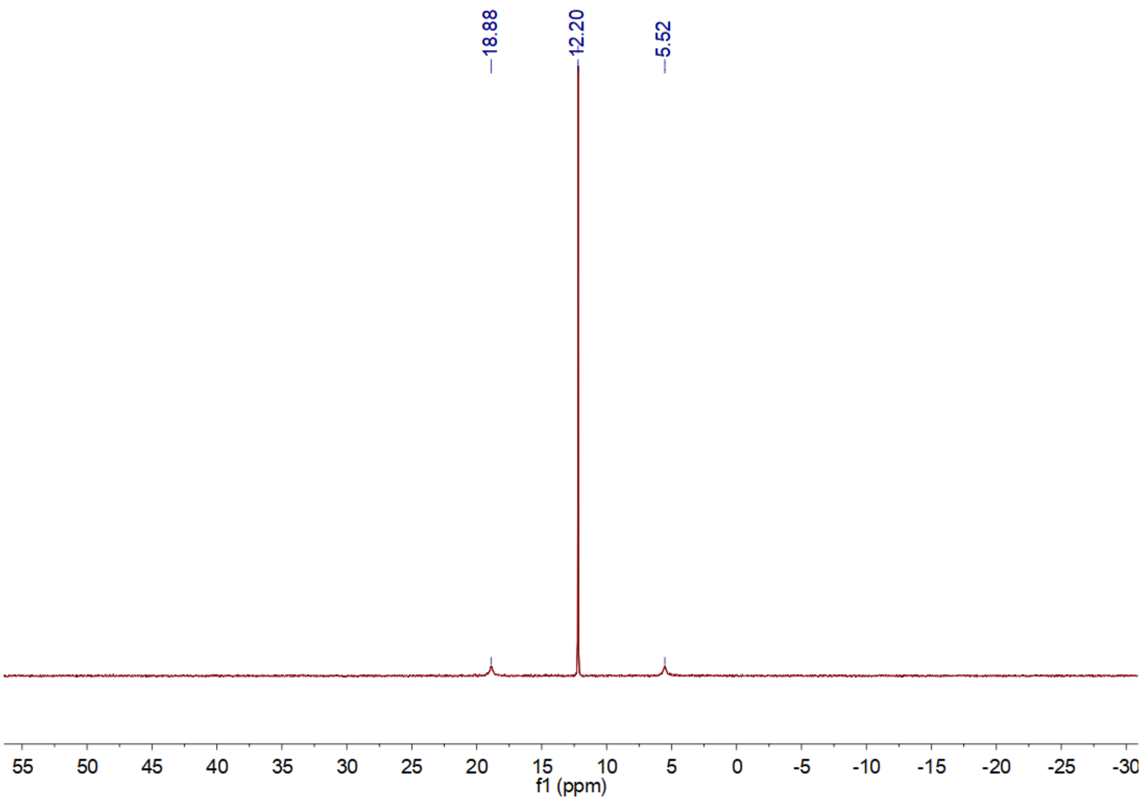

Supplementary Fig. 24 $^{31}P\{^{1}H\}$ NMR spectrum (202 MHz, CD_2Cl_2, 298 K) of rhomboid **9**.

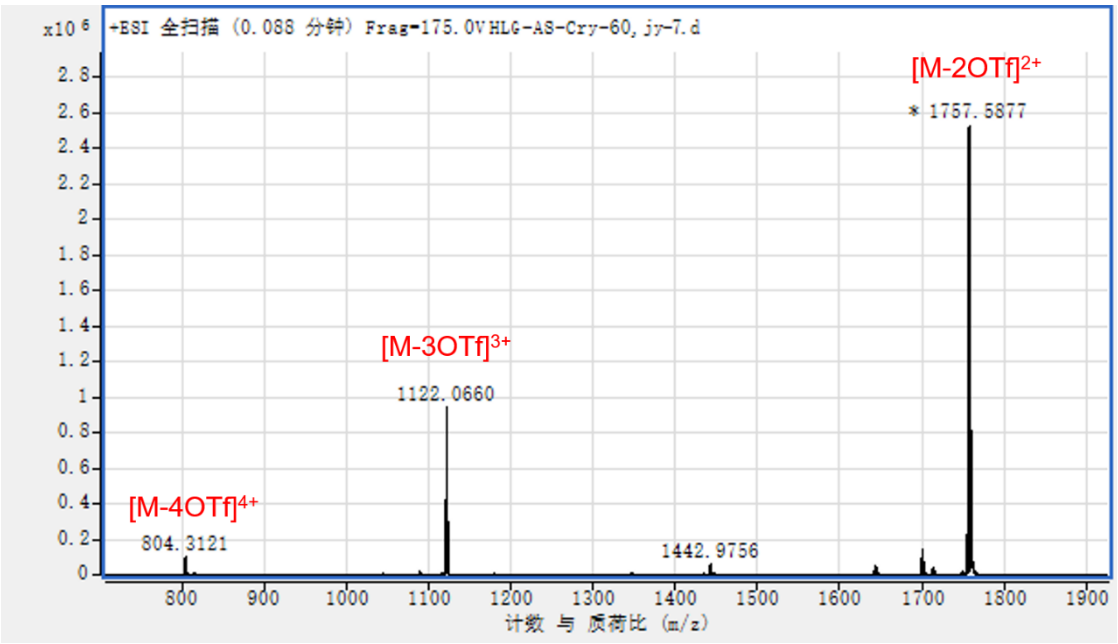

Supplementary Fig. 25 Electrospray ionization mass spectrum of rhomboid **9**.

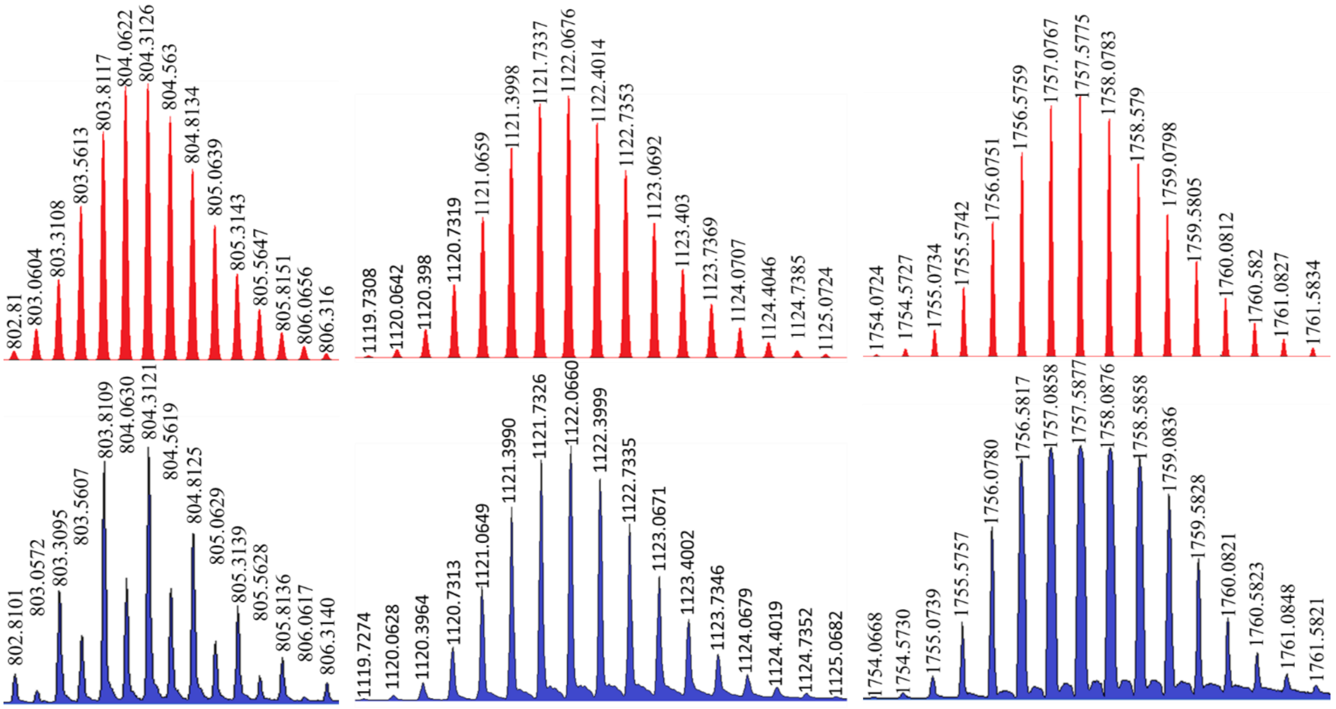

Supplementary Fig. 26 Calculated (red) and experimental (blue) electrospray ionization mass spectrum of rhomboid **9**.

5. Synthesis of norbornene-functionalized hexagon 10

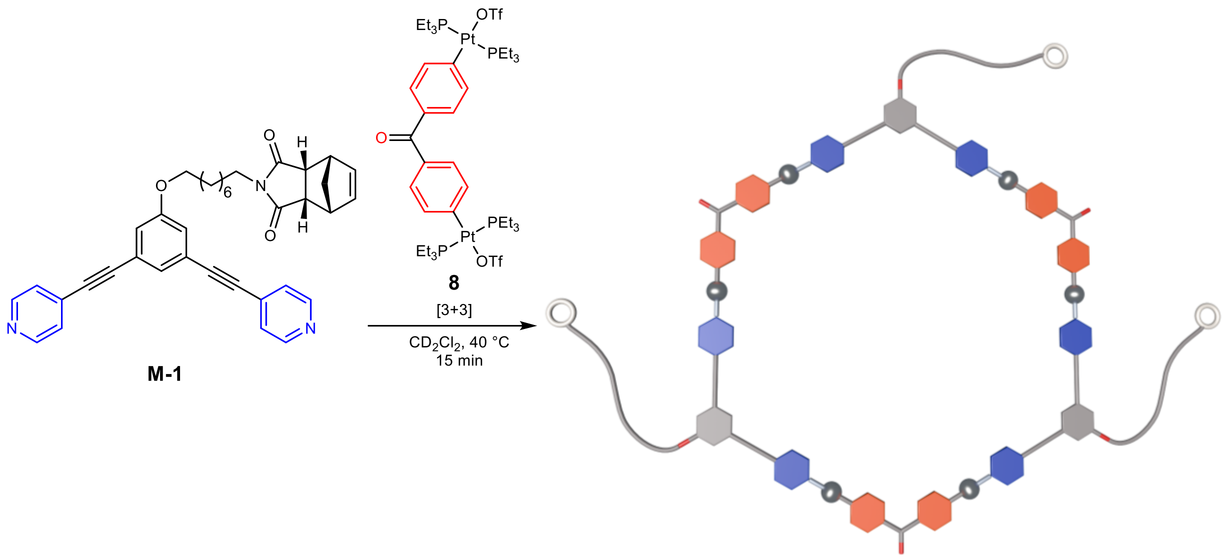

Monomer **M-1** (3.42 mg, 0.006 mmol) and 120° diplatinum acceptor **8** (8.02 mg, 0.006 mmol) were dissolved in 0.6 mL of CD_2Cl_2 in a vial. The reaction mixture was heated at 40 °C for 15 min to give a homogeneous solution of metallacycle **10** that was directly used for analysis. The ^{1}H NMR spectrum of metallacycle **10** is shown in Supplementary Fig. 27. ^{1}H NMR (500 MHz, CD_2Cl_2, 298 K) δ 8.72 (d, J = 6 Hz, 12H), 7.83 (d, J = 6 Hz, 12H), 7.57–7.51 (m, 27H), 7.25 (d, J = 1 Hz, 6H), 6.08 (t, J = 2 Hz, 6H), 4.04 (t, J = 6 Hz, 6H), 3.35–3.32 (m, 6H), 3.29 (t, J = 7 Hz, 6H), 3.23–3.21 (m, 6H), 1.85–1.79 (m, 6H), 1.70 (dt, J = 9, 2 Hz, 3H), 1.54 (dt, J = 9, 2 Hz, 3H), 1.49–1.44 (m, 12H), 1.40–1.35 (m, 72H), 1.32–1.28 (m, 18H), 1.18–1.12 (m, 108H). The $^{31}P\{^{1}H\}$ NMR spectrum of metallacycle **10** is shown in Supplementary Fig. 28. $^{31}P\{^{1}H\}$ NMR (202 MHz, CD_2Cl_2, 298 K) δ 12.94 (s, ^{195}Pt satellites, $J_{Pt\text{-}P}$ = 2644 Hz). HRMS (ESI/Q-TOF) of **10** is shown in Supplementary Fig. 29. m/z: [M – 6OTf]$^{6+}$ calcd for $C_{222}H_{309}N_9O_{12}P_{12}Pt_6$, 806.31; found, 806.3065. m/z: [M – 5OTf]$^{5+}$ calcd for $C_{223}H_{309}F_3N_9O_{15}P_{12}Pt_6S$, 997.3625; found, 997.3521. m/z: [M – 4OTf]$^{4+}$ calcd for $C_{224}H_{309}F_6N_9O_{18}P_{12}Pt_6S_2$, 1283.9412; found, 1283.9280. m/z: [M – 3OTf]$^{3+}$ calcd for $C_{225}H_{309}F_9N_9O_{21}P_{12}Pt_6S_3$, 1761.5724; found, 1761.4678.

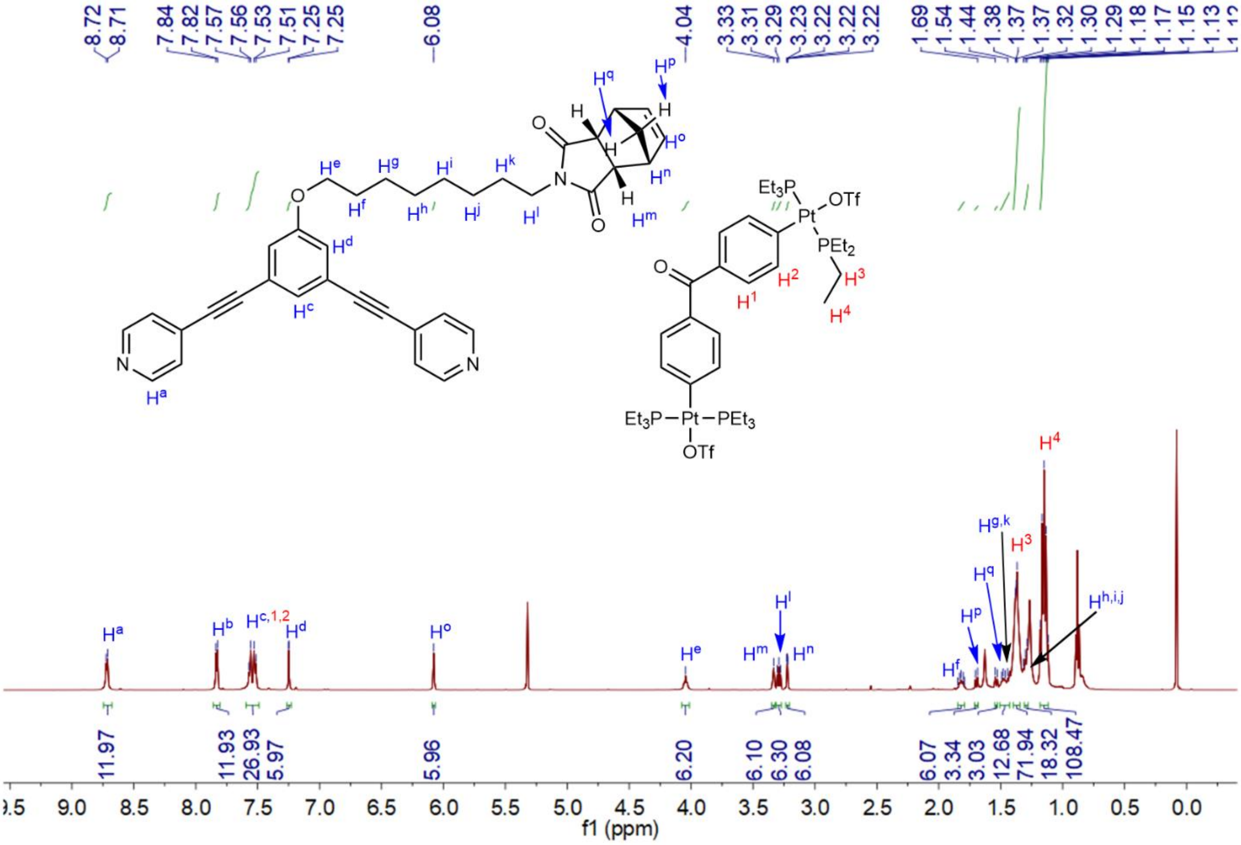

Supplementary Fig. 27 ^{1}H NMR spectrum (500 MHz, CD_2Cl_2, 298 K) of hexagon **10**.

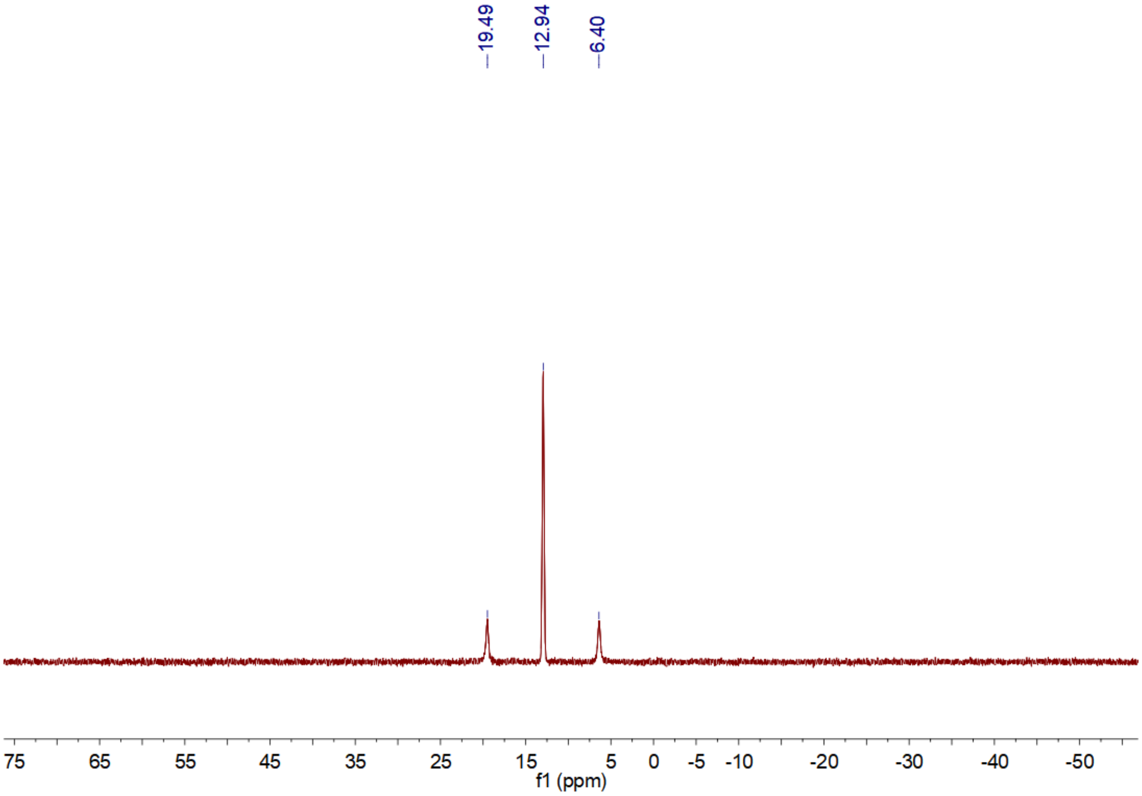

Supplementary Fig. 28 $^{31}P\{^{1}H\}$ NMR spectrum (202 MHz, CD_2Cl_2, 298 K) of hexagon **10**.

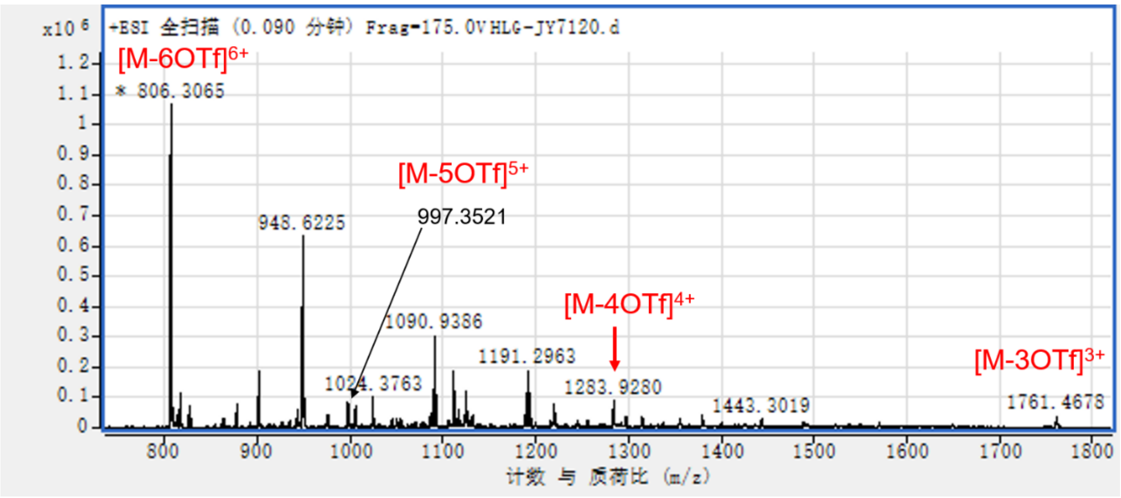

Supplementary Fig. 29 Electrospray ionization mass spectrum of hexagon **10**.

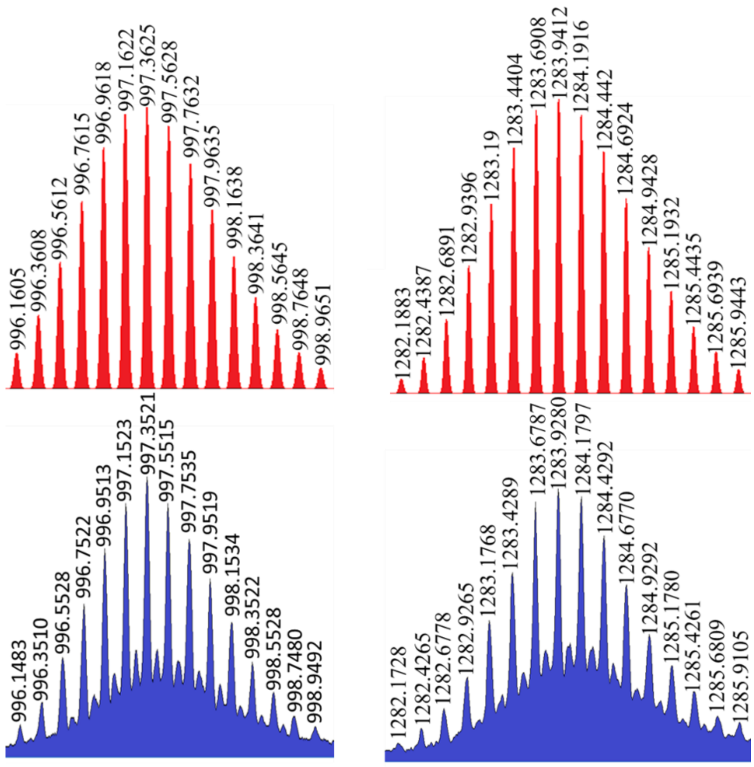

Supplementary Fig. 30 Calculated (red) and experimental (blue) electrospray ionization mass spectrum of hexagon **10**.

6. Preparation of covalent polymers CPs

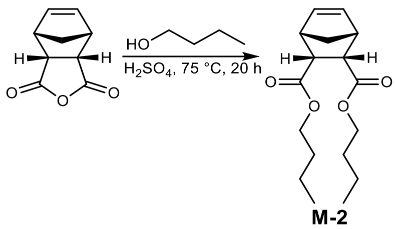

To a 100 mL round-bottomed flask was added *endo*-5-norbornene-2,3-dicarboxylic anhydride (2.00 g, 12.20 mmol), followed by 20 mL of anhydrous 1-butanol. To this was added 4 drops of concentrated sulfuric acid, and the resulting mixture was stirred under air at 75 °C for 20 h. The colorless solution was allowed to cool to room temperature and concentrated in vacuo. The crude oil was redissolved in 100 mL CH_2Cl_2. The mixture was washed with water and brine, and then dried over anhydrous Na_2SO_4. The solution was concentrated and the resulting residue was purified by flash column chromatography (ethyl acetate/petroleum ether, 1:50 *v*/*v*) to afford compound **M-2** as colorless oils. ^1H NMR (500 MHz, $CDCl_3$, 298 K) δ 6.25 (d, *J* = 2 Hz, 2H), 3.99 (ddt, *J* = 38, 11, 7 Hz, 4H), 3.29–3.24 (m, 2H), 3.15 (m, 2H), 1.60–1.55 (m, 4H), 1.54 (dt, *J* = 9, 2 Hz, 1H), 1.46 (dt, *J* = 9, 2 Hz, 1H), 1.38–1.32 (m, 4H), 0.92 (t, *J* = 7 Hz, 6H).

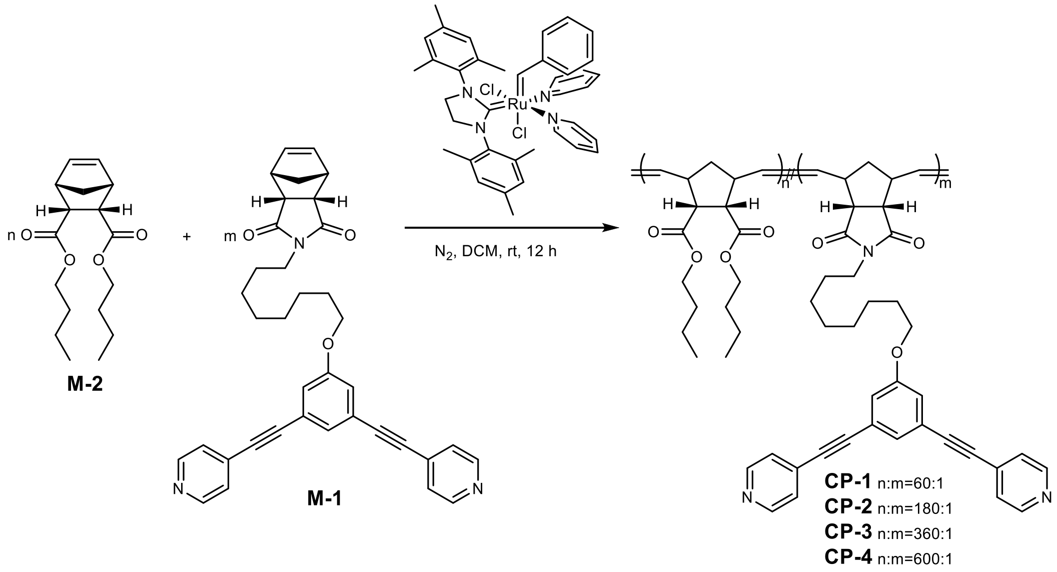

Preparation of pyridine-modified 2nd generation Grubbs' catalyst[S5]: The 2nd generation Grubbs' catalyst (250.00 mg, 0.294 mmol) was placed in a Schlenk flask and pyridine (2.45 mL, 30.413 mmol) was added under N_2 atmosphere. After the mixture solution was stirred for 15 min at room temperature, the solution color changed from red to green. The resulted solution was poured into hexane and the

precipitate was then collected by centrifugation. After it was dried under vacuum, the pyridine-modified Grubbs' catalyst was obtained as a green solid.

Preparation of CP-1: An oven-dried vial was charged with norbornenyl *endo*-di-n-butyl ester **M-2** (900.00 mg 3.057 mmol), compound **M-1** (29.06 mg, 0.051 mmol) and a stirring bar. The vial was degassed, and 3 mL of degassed anhydrous CH_2Cl_2 was then added via a syringe under nitrogen atmosphere to dissolve the two monomers. A solution of pyridine-modified Grubbs catalyst (11.00 mg, 0.015 mmol) in 3 mL of degassed anhydrous CH_2Cl_2 was injected into the solution of monomers to initiate the polymerization. The solution was stirred for 12 h at room temperature and quenched by the addition of 0.2 mL of neat ethyl vinyl ether. After the mixture was stirred for additional 60 min, it was added dropwise to 1.0 L of methanol under rapid stirring to produce a reddish brown precipitate. The suspension was then centrifuged. The solid was collected and dried under vacuum for 48 h to afford **CP-1** (813.00 mg, 87%). The ^1H NMR spectrum of **CP-1** is shown in Supplementary Fig. 31. The GPC curve of **CP-1** is shown in Supplementary Fig. 32.

Copolymers **CP-2**, **CP-3**, and **CP-4** were synthesized by the method similar to that for **CP-1**.

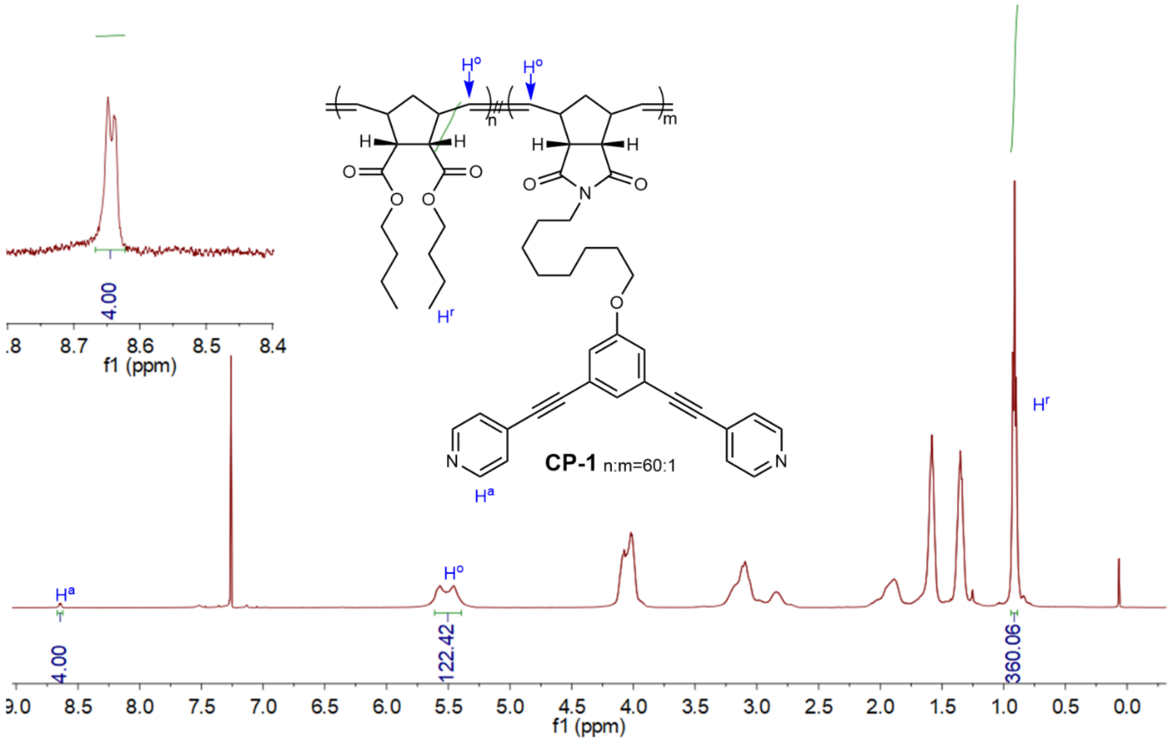

Supplementary Fig. 31 ^1H NMR spectrum (500 MHz, $CDCl_3$, 298 K) of **CP-1**.

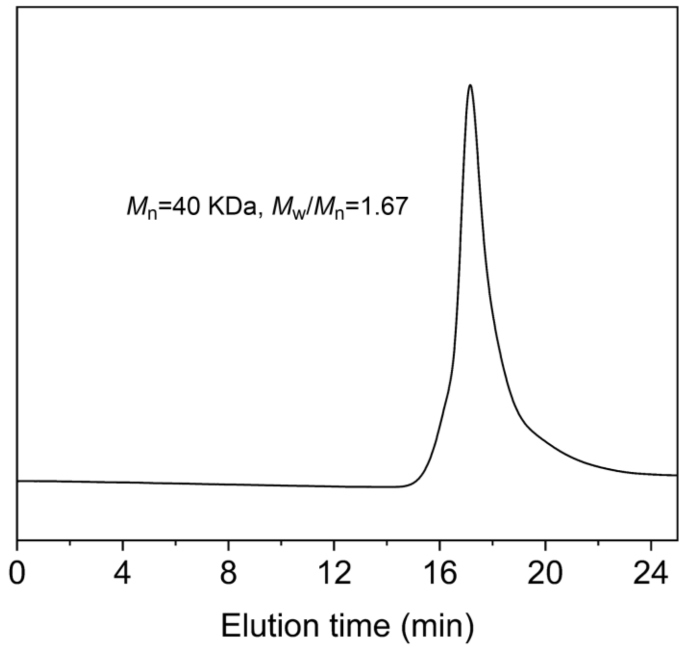

Supplementary Fig. 32 GPC elution curve of **CP-1** with THF as the eluent and polystyrene (PS) as the standard.

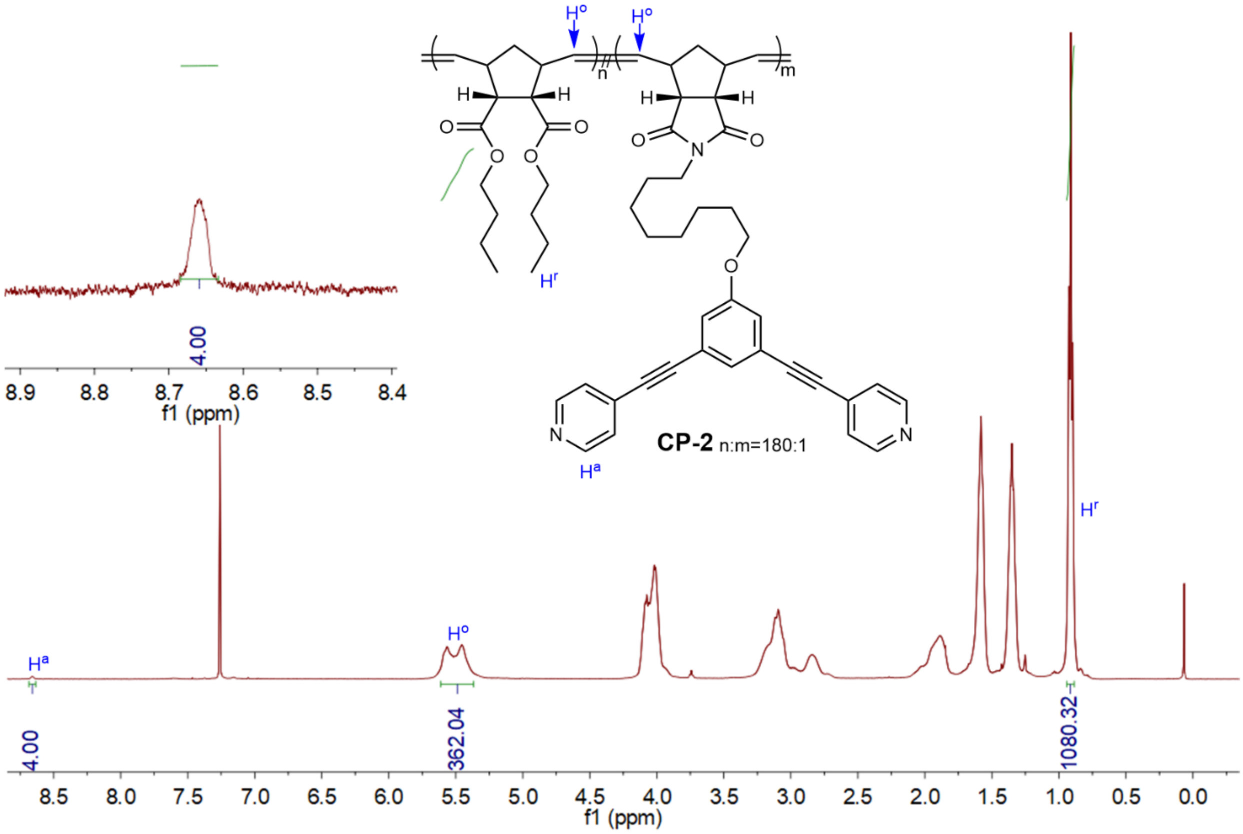

Supplementary Fig. 33 ^{1}H NMR spectrum (500 MHz, $CDCl_3$, 298 K) of **CP-2**.

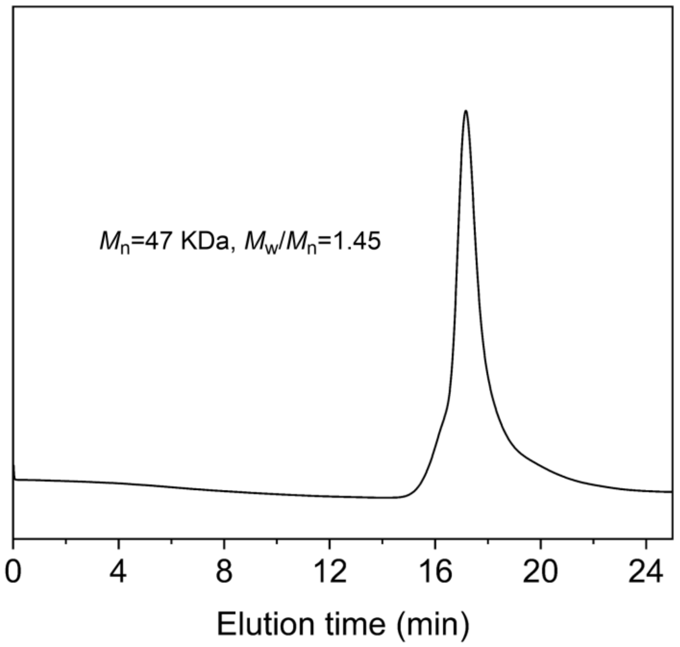

Supplementary Fig. 34 GPC elution curve of **CP-2** with THF as the eluent and polystyrene (PS) as the standard.

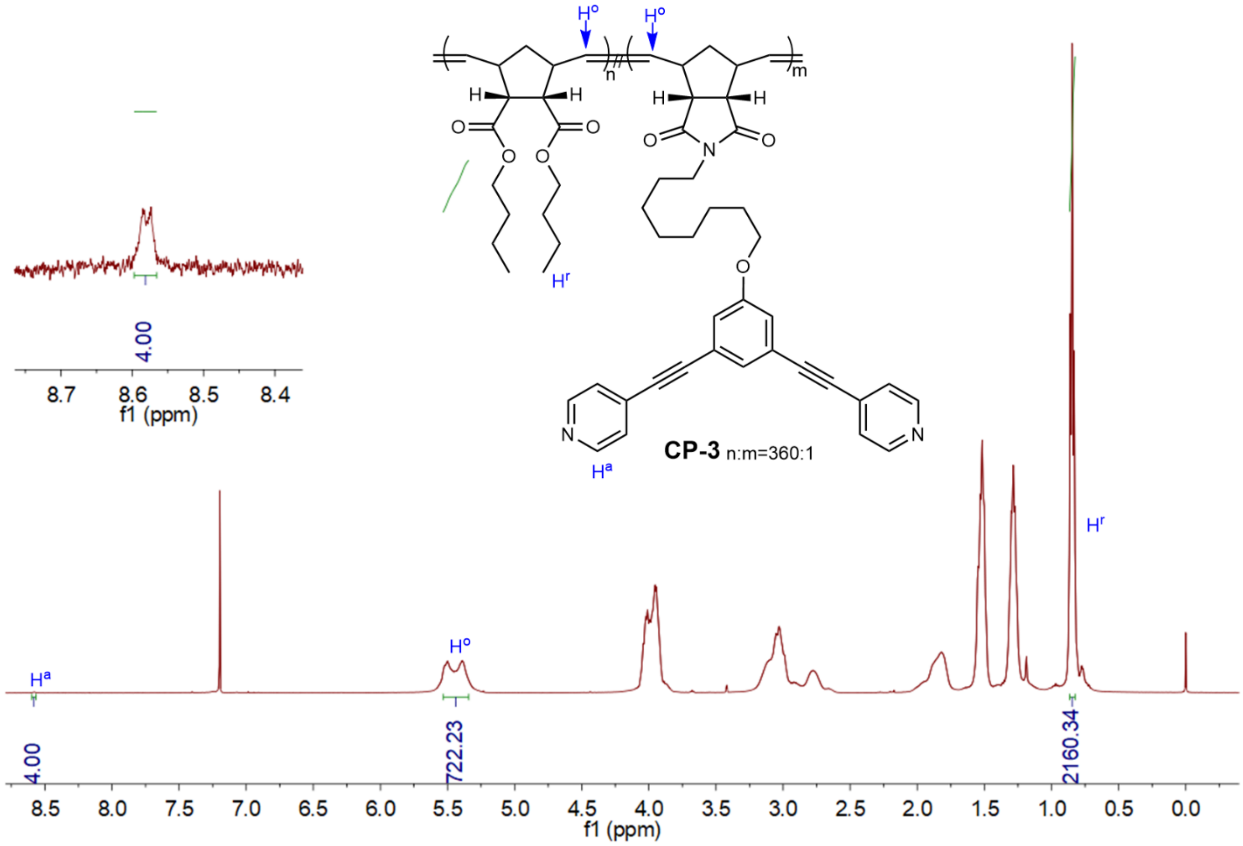

Supplementary Fig. 35 ^{1}H NMR spectrum (500 MHz, $CDCl_3$, 298 K) of **CP-3**.

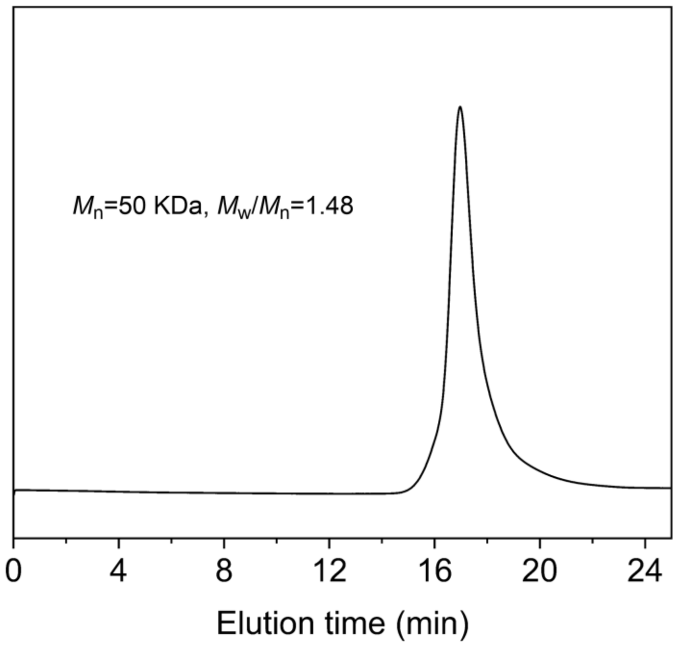

Supplementary Fig. 36 GPC elution curve of **CP-3** with THF as the eluent and polystyrene (PS) as the standard.

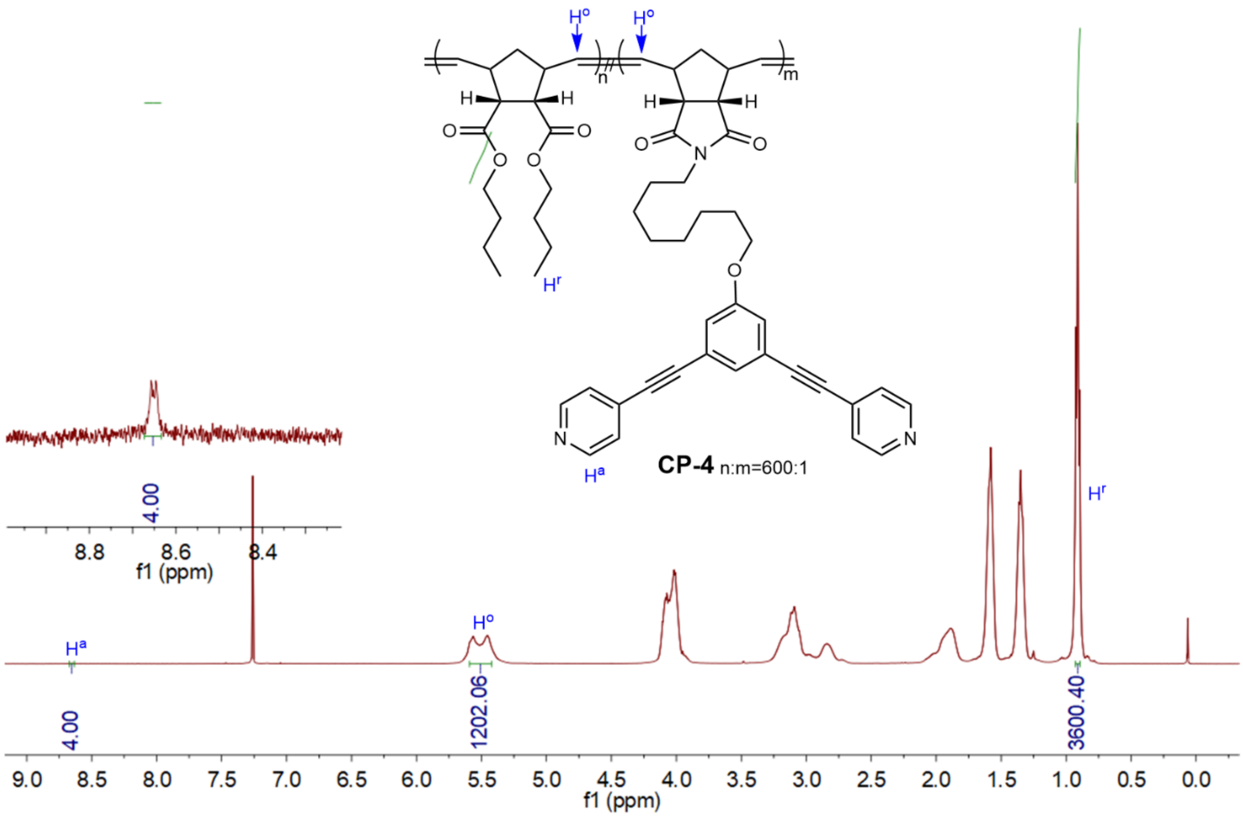

Supplementary Fig. 37 ^{1}H NMR spectrum (500 MHz, $CDCl_3$, 298 K) of **CP-4**.

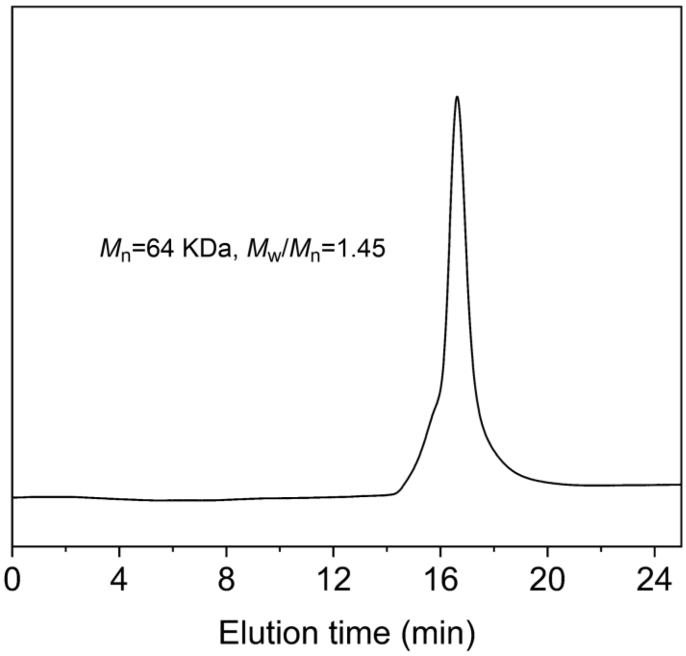

Supplementary Fig. 38 GPC elution curve of **CP-4** with THF as the eluent and polystyrene (PS) as the standard.

Supplementary Table 1. General Information of **CP-1**, **CP-2**, **CP-3**, **and CP-4**.

Sample	M_n (KDa)	M_w (KDa)	M_w/M_n	Mol% of dipyridine modified units
**CP-1**	40	67	1.67	1.64
**CP-2**	47	68	1.45	0.55
**CP-3**	50	74	1.48	0.28
**CP-4**	64	93	1.45	0.17

7. Preparation of metallacycle-crosslinked polymer networks CP-*n*Rs

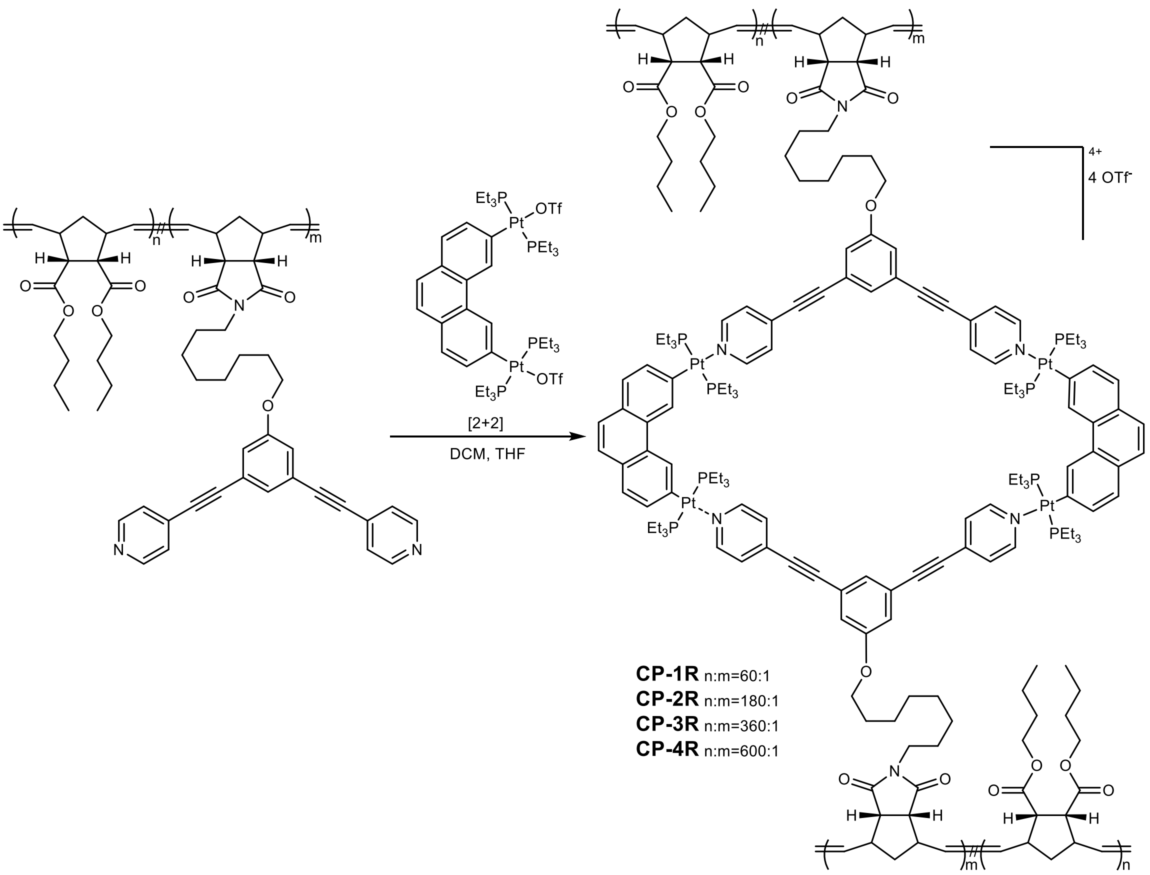

Covalent polymer **CP-1** (300.00 mg, with 0.016 mmol pyridine side group) was dissolved in 1 mL of THF in a vial, the 60° diplatinum acceptor **7** (21.39 mg, 0.016 mmol) in 0.4 mL of CH_2Cl_2 was added into the solution and stirred for 15 min. The obtained solution was poured onto a rectangular Teflon mold and the solvents were slowly evaporated at room temperature to obtain a dry thin film of **CP-1R** for various tests.

CP-2R, **CP-3R**, and **CP-4R** were prepared by the method similar to that for **CP-1R**.

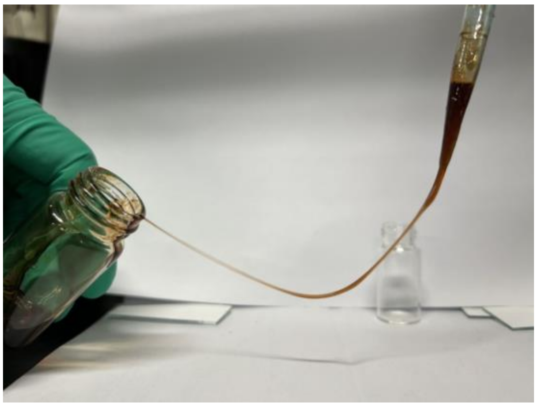

Supplementary Fig. 39 Photo of silk drawn from a solution of **CP-3R**.

8. Preparation of metallacycle-crosslinked polymer networks CP-*n*Hs

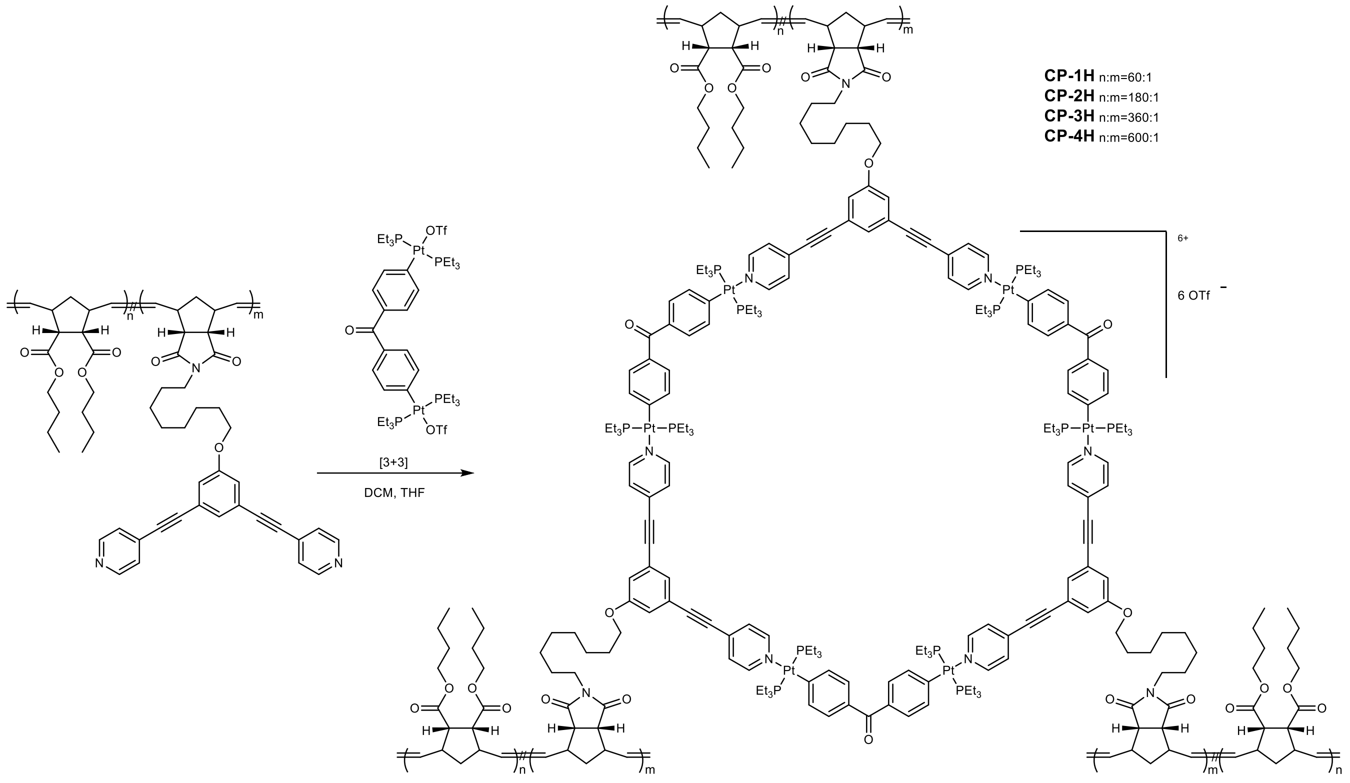

Covalent polymer **CP-1** (300.00 mg, with 0.016 mmol pyridine side group) was dissolved in 1 mL of THF in a vial, the 120° diplatinum acceptor **8** (21.46 mg, 0.016 mmol) in 0.4 mL of CH_2Cl_2 was added into the solution and stirred for 15 min. The obtained solution was poured onto a rectangular Teflon mold and the solvents were slowly evaporated at room temperature to obtain a dry thin film of **CP-1H** for various tests. **CP-2H**, **CP-3H**, and **CP-4H** were prepared by the method similar to that for **CP-1H**.

9. TGA of metallacycle-crosslinked polymer networks

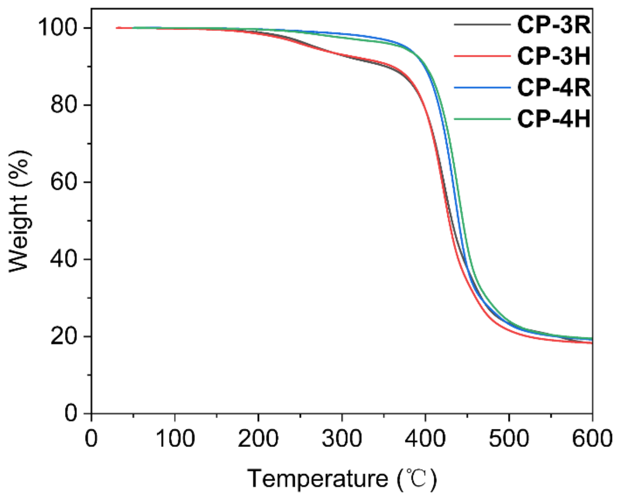

Supplementary Fig. 40 TGA curves of **CP-*n*R**s and **CP-*n*H**s under N_2 flow (50 mL/min) with a heating rate of 20 °C/min.

10. Possible incomplete assemblies during the self-assembly of dipyridine units in polymers

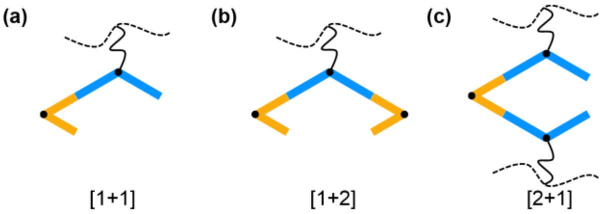

Supplementary Fig. 41 The three possible incomplete assemblies formed by the self-assembly of 60° diplatinum acceptor **7** and dipyridine units in polymers. (The yellow bar represents compound **7**, and the blue bar represents the pendant dipyridine units). **a** [1+1] self-assembly of one dipyridine unit and one 60° diplatinum acceptor. **b** [1+2] self-assembly of one dipyridine unit and two 60° diplatinum acceptors. **c** [2+1] self-assembly of two dipyridine units and one 60° diplatinum acceptor.

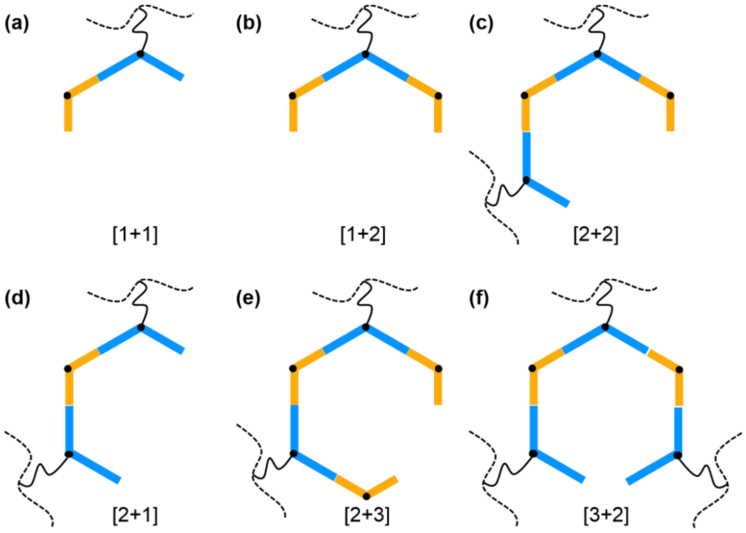

Supplementary Fig. 42 The three possible incomplete assemblies formed by the self-assembly of 120° diplatinum acceptor **8** and dipyridine units in polymers. (The yellow bar represents compound **8**, and the blue bar represents the pendant dipyridine units). **a** [1+1] self-assembly of one bipyridine unit and one 120° diplatinum acceptor. **b** [1+2] self-assembly of one bipyridine unit and two 120° diplatinum acceptors. **c** [2+2] self-assembly of two bipyridine units and two 60° diplatinum acceptors. **d** [2+1] self-assembly of two bipyridine units and one 120° diplatinum acceptor. **e** [2+3] self-assembly of two bipyridine units and three 120° diplatinum acceptors. **f** [3+2] self-assembly of three bipyridine units and two 120° diplatinum acceptors.

11. Preparation of control ^{con}CP-3R and ^{con}CP-3H

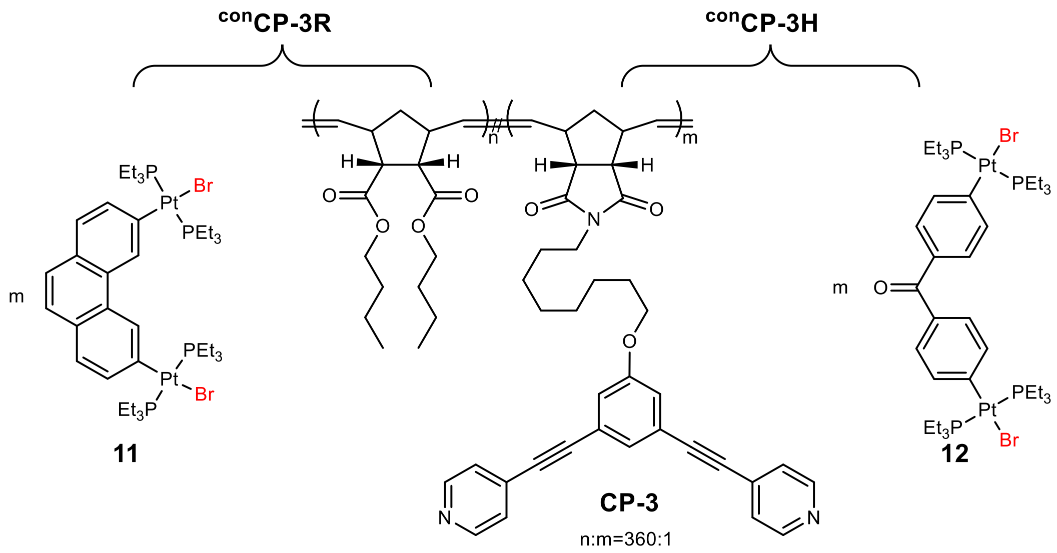

Supplementary Fig. 43 Constitutions of control **^{con}CP-3R** and **^{con}CP-3H**.

12. Mechanical properties of CP-3, CP-3R, CP-4R, CP-3H, CP-4H and the controls

Supplementary Table 2. Mechanical properties of **CP-3**, **CP-3R**, **CP-4R**, **CP-3H**, **CP-4H** and the controls.

Sample	Yield stress (MPa)	Strain at break (%)	Young's modulus (MPa)	Toughness (MJ/m^3)
**CP-3**	4.85	675	106.01	43.4
**CP-3R**	33.62	698	653.16	154.02
**CP-4R**	27.54	1011	401.69	199.48
**CP-3H**	21.18	944	400.50	155.12
**CP-4H**	21.44	621	364.74	86.63
**^{con}CP-3R**	4.84	791	95.89	29.44
**^{con}CP-3H**	4.87	680	98.45	24.68

13. The storage modulus of CP-3, CP-3R and CP-3H from DMA

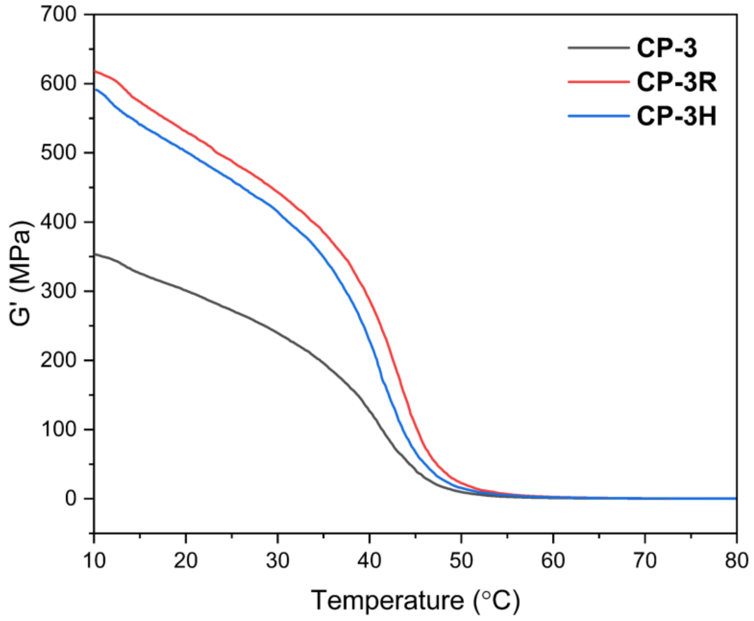

Supplementary Fig. 44 The storage modulus of **CP-3**, **CP-3R** and **CP-3H**.

14. The swelling of CP-3R and CP-3H, and the dissolution of CP-3

The dry films of **CP-3R**, **CP-3H** and **CP-3** were mixed with 3 mL of 1,2-dichloroethane, respectively. The appearances of these films were recorded at different times.

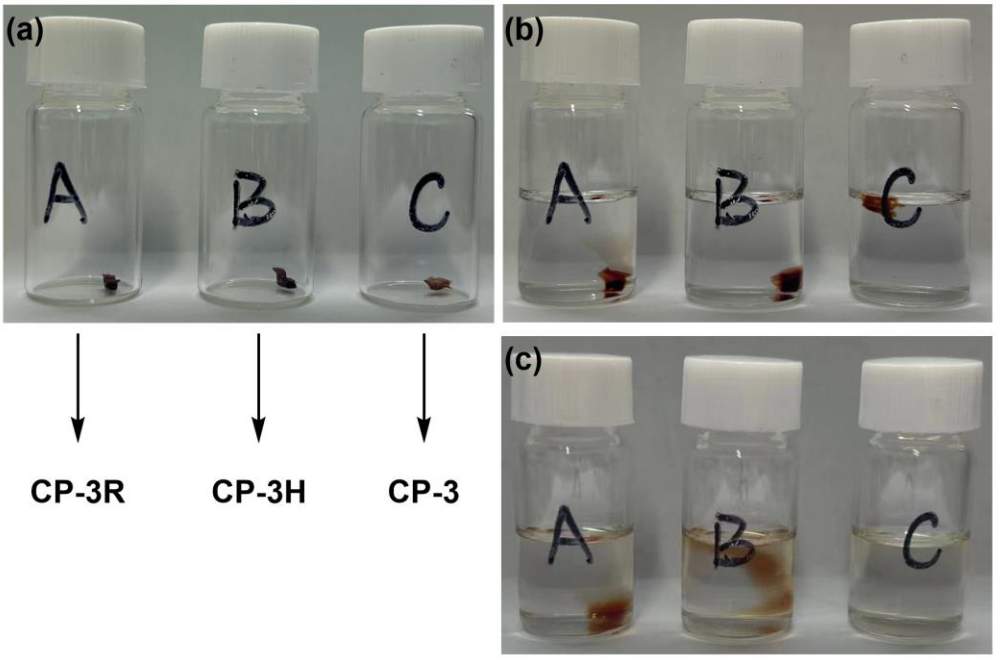

Supplementary Fig. 45 The images of the swelling of **CP-3R** and **CP-3H**, and the dissolution of **CP-3**. **a** The films before mixing with 1,2-dichloroethane. **b** The films mixed with 1,2-dichloroethane for 5 min. **c** The films mixed with 1,2-dichloroethane for 12 h.

15. Morphologies of the xerogels of CP-3R and CP-3H

The xerogels **CP-3R** and **CP-3H** were obtained through the following method:

A dry thin film of **CP-3R** or **CP-3H** (3 mg) was placed in a centrifuge tube, followed by adding 3 mL of 1,2-dichloroethane, and the film was swollen after 12 hours. The xerogel was obtained from the swollen film after freeze-drying.

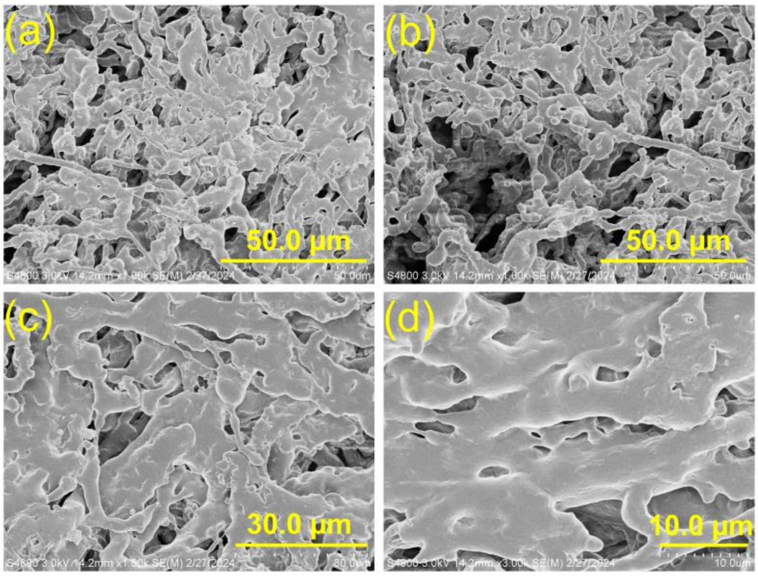

Supplementary Fig. 46 SEM images of the xerogel **CP-3R**. SEM images with a scale bar of 50.0 μm (**a**, **b**), 30.0 μm (**c**), and 10.0 μm (**d**), respectively.

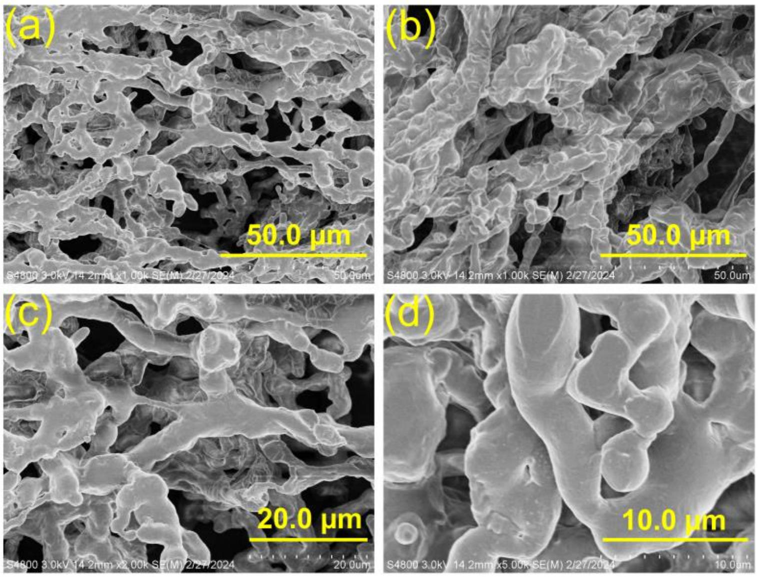

Supplementary Fig. 47 SEM images of the xerogel **CP-3H**. SEM images with a scale bar of 50.0 μm (**a**, **b**), 20.0 μm (**c**), and 10.0 μm (**d**), respectively.

16. Stimuli-responsive properties of metallacycle-crosslinked polymer networks

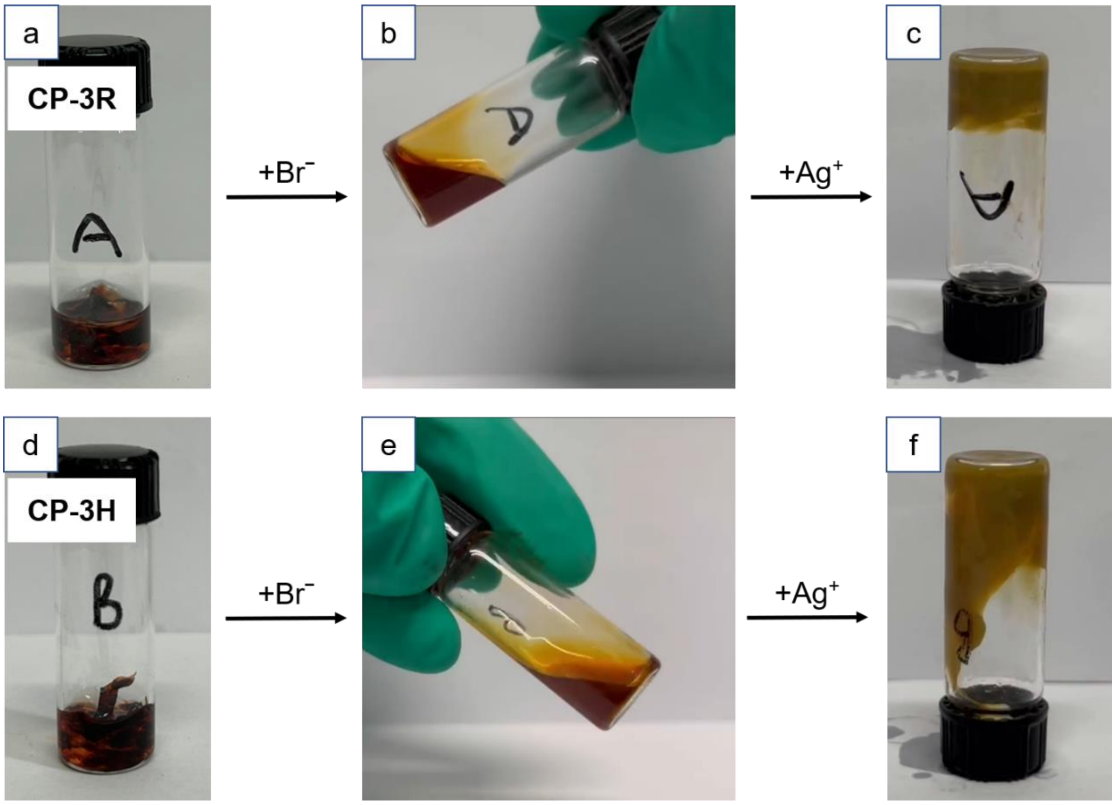

Supplementary Fig. 48 The stimuli-responsive behavior of **CP-3R** and **CP-3H**. **a** Partially dissolved cut of **CP-3R**. **b** THF solution of **CP-3R** after addition of TBABr. **c** THF solution of **CP-3R** after addition of TBABr and AgOTf in sequence. **d** Partially dissolved cut of **CP-3H**. **e** THF solution of **CP-3H** after addition of TBABr. **f** THF solution of **CP-3H** after aaddition of TBABr and AgOTf in sequence.

17. Mechanical properties of CP-3R + Br^-, CP-3R + Br^- + Ag^+, CP-3H + Br^- and CP-3H + Br^-+ Ag^+

Supplementary Table 3. Mechanical properties of **CP-3R + Br^-, CP-3R + Br^- + Ag^+, CP-3H + Br^- and CP-3H + Br^-+ Ag^+.**

Sample	Yield stress (MPa)	Strain at break (%)	Young's modulus (MPa)	Toughness (MJ/m^3)
**CP-3R + Br^-**	7.43	1163	136.17	97.14
**CP-3R + Br^- + Ag^+**	16.62	614	407.79	81.43
**CP-3H + Br^-**	8.79	1196	139.66	100.89
**CP-3H + Br^- + Ag^+**	13.93	652	290.9	66.45

18. Single-crystal X-ray structure of 9

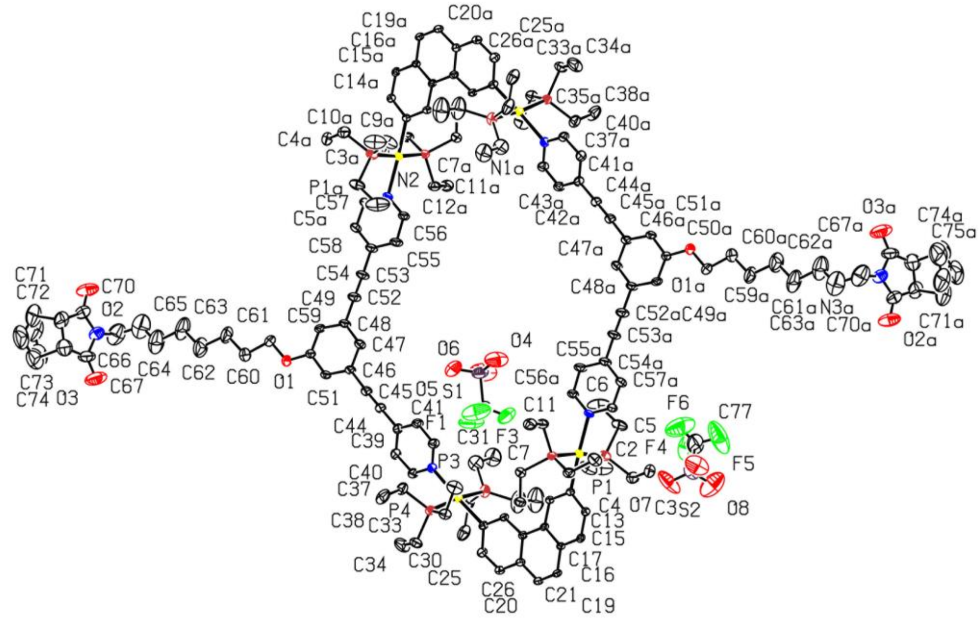

Supplementary Fig. 49 Single-crystal X-ray structure of **9**

Supplementary Table 4. Crystal data and structure refinement for compound **9**.

Deposition number	**2297067**
Empirical formula	$C_{77}H_{103}F_6N_3O_9P_4Pt_2S_2$
Formula weight	1906.80
Temperature/K	170.0
Crystal system	triclinic
Space group	*P*-1
a/Å	10.7770(5)
b/Å	18.1721(10)
c/Å	25.1254(13)
α/°	87.498(2)
β/°	79.287(2)
γ/°	84.285(2)
Volume/Å^3	4809.2(4)
Z	2
ρ_{calc} g/cm^3	1.317
μ/mm^{-1}	3.074
F(000)	1920.0
Crystal size/mm^3	0.49 × 0.38 × 0.32
Radiation	MoKα (λ = 0.71073)
2θ range for data collection/°	3.948 to 54.458
Index ranges	$-13 \leq h \leq 13, -23 \leq k \leq 23, -32 \leq l \leq 32$
Reflections collected	119449
Independent reflections	21305 [R_{int} = 0.0737, R_{sigma} = 0.0506]
Data/restraints/parameters	21305/299/979
Goodness-of-fit on F^2	1.044
Final R indexes [I>=2σ (I)]	R_1 = 0.0473, wR_2 = 0.1269
Final R indexes [all data]	R_1 = 0.0569, wR_2 = 0.1338
Largest diff. peak/hole / e Å^{-3}	2.19/-2.29

19. Supplementary References

1. Ignasik, M. et al. Design, Synthesis and Evaluation of Novel 2-(Aminoalkyl)-isoindoline-1,3-dione Derivatives as Dual-Binding Site Acetylcholinesterase Inhibitors. *Arch. Pham.* **345**, 509-516 (2012).
2. Yang, H.-B., Ghosh, K., Arif, A. M. & Stang, P. J. The Synthesis of New 60° Organometallic Subunits and Self-Assembly of Three-Dimensional M3L2 Trigonal-Bipyramidal Cages. *J. Org. Chem.* **71**, 9464-9469 (2006).
3. Yang, H.-B. et al. A Highly Efficient Approach to the Self-Assembly of Hexagonal Cavity-Cored Tris[2]pseudorotaxanes from Several Components via Multiple Noncovalent Interactions. *J. Am. Chem. Soc.* **129**, 14187-14189 (2007).
4. Cater, H. L., Balynska, I., Allen, M. J., Freeman, B. D. & Page, Z. A. User Guide to Ring-Opening Metathesis Polymerization of endo-Norbornene Monomers with Chelated Initiators. *Macromolecules* **55**, 6671-6679 (2022).
5. Love, J. A., Morgan, J. P., Trnka, T. M. & Grubbs, R. H. A Practical and Highly Active Ruthenium-Based Catalyst that Effects the Cross Metathesis of Acrylonitrile. *Angew. Chem. Int. Ed.* **41**, 4035-4037 (2002).
